# Hypersensitivity of chitin degradation to initial species densities due to monomer diffusion

**DOI:** 10.1073/pnas.2512676123

**Published:** 2026-01-05

**Authors:** Sammy Pontrelli, Ghita Guessous, Julian Trouillon, Aswin Krishna, Terence Hwa, Uwe Sauer

**Affiliations:** ^a^Institute of Molecular Systems Biology, ETH Zürich, Zürich 3001, Switzerland; ^b^Department of Biology, University of Leuven, Leuven 3001, Belgium; ^c^Flanders Institute for Biotechnology (VIB), Center for Microbiology, Leuven 3001, Belgium; ^d^Department of Physics, University of California, San Diego, La Jolla, CA 92093-0319

**Keywords:** microbial ecology, microbial competition, quantitative biology, microbial interactions, polysaccharide degradation

## Abstract

Resource competition strongly shapes microbial community dynamics and functionality. In polysaccharide-degrading communities, primary degraders release hydrolytic enzymes, whereas exploiters consume released products without producing enzyme themselves. We investigate the competitive strategies employed by marine chitin degraders and N-acetylglucosamine (GlcNAc) exploiters, revealing various mechanisms that impact community viability and growth dynamics. In addition to direct competition strategies such as antibiotic secretion or cell aggregation on chitin particles (which helps monopolize enzyme access), exploiters also inhibit degraders by diverting limiting GlcNAc flux during the early stages of particle degradation. This critical phase requires degraders to overcome the diffusive loss of GlcNAc to sustain their chitinase production. Through quantitative measurements and modeling, we demonstrate that nutrient competition among species and nutrient loss through diffusion during the initial stages of community dynamics strongly influence the long-term success of the community. The initial community composition dictates the former mechanisms, while the latter is closely related to particle size, both of which have profound implications for environmental carbon cycling. The resulting hypersensitivity of the community is analogous to the Allee effect observed in population biology, where the outcomes—in our case polymer degradation—are heavily dependent on starting conditions. This study sheds light on how metabolic competition in the early phases of particle degradation governs species interactions, resource partitioning, and overall community viability, even under identical environmental and genetic conditions.

## Significance

Competitive interactions shape key metabolic processes in microbial communities. As a model to study these interactions, we examine microbes that degrade chitin—one of the most prevalent polysaccharides in nature. Our findings demonstrate that growth dynamics become highly sensitive to the initial ratios of bacterial species competing for chitin degradation products. This sensitivity arises from two main factors: diffusive loss of monomers and competition between chitin degrading species and nondegraders that intercept these monomers. Even small populations of nondegraders can significantly undermine the resources available to chitin-degraders, potentially inhibiting community growth entirely. These findings illustrate how early-stage interactions govern microbial community functionality and suggest that similar sensitivity to initial conditions could affect outcomes in various environmental contexts.

Resource competition and sharing are important drivers of interspecies dynamics in microbial communities (1–7). In both laboratory experiments (3, 6, 8, 9) and complex environments ranging from the ocean to the mammalian gut (10), microbes with similar metabolic capabilities often compete for resources leading to species’ exclusion; yet some communities can sustain metabolically similar species coexisting on the same resources (11–16). This raises questions about other contextual factors that influence species’ outcomes beyond differences in resource affinities, including spatial structure (17, 18), biological warfare (19, 20), and sensitivity to initial community composition (21, 22). To understand how metabolically similar species coexist or exclude one another, it is essential to disentangle the relationship between resource partitioning, competitive strategies, and environmental context.

Competition for publicly available resources is particularly prevalent in polysaccharide-degrading communities, with important implications for human health (23), biotechnology (24), and biogeochemical cycling (25). These communities are characterized by the presence of specialized degraders that release hydrolytic enzymes extracellularly to break down polysaccharides into transportable monomers and oligomers (26, 27). The extracellular degradation of polysaccharides generates publicly available mono- and oligomers that can be utilized not only by the degraders themselves but also by exploiters that do not produce their own hydrolytic enzymes (27–30). The competitive dynamics between exploiters and degraders play a significant role in shaping population dynamics and polysaccharide degradation rates (3, 21, 31, 32), which has fundamental consequences for community function. The presence of exploiters may lead to diminished returns for degraders from their investment in hydrolytic enzymes, potentially resulting in the extinction of degraders, and, in extreme cases, the collapse of the community. To avert such dire situations, degraders implement various protective strategies, each with their own limitations. One such strategy is to increase enzyme expression to enhance carbon flow (33, 34); however, this approach is limited by the degrader’s ability to intake consumable monomers and oligomers that have been siphoned off by the exploiters. To enhance access to monomers in spatially structured environments, both degraders and exploiters can aggregate on polysaccharide surfaces (17, 35–37) or rapidly disperse to new nutrient patches (35, 36, 38, 39). Additionally, to minimize diffusive loss, they may employ outer membrane-bound hydrolytic enzymes for selfish oligomer uptake (28, 40–42). The key question revolves around understanding the potential advantages and costs associated with the various strategies employed by these competing species.

Here, we investigate the competitive strategies of degraders and exploiters in a model marine chitin-degrading community. Chitin, the most abundant polysaccharide in the ocean (43), exists primarily in an insoluble form, allowing us to consider the role of spatial structure and thus of microbial attachment and aggregation in the competitive dynamics. This has implications for carbon cycling in marine ecosystems as well as other microbial systems that degrade insoluble polysaccharides (26, 44, 45). To identify instances of direct competition for the chitin monomer N-acetylglucosamine (GlcNAc), we screened pairwise cocultures of 14 natural bacterial isolates (*SI Appendix*, Table S1) (12, 45), previously shown to recapitulate population dynamics of chitin-degrading consortia (3). Through a detailed characterization of five specific pairs, we aimed to discern the key factors influencing competition between degraders and exploiters, along with their growth dynamics. In some cases, antibiotic production enabled degraders to protect themselves from exploiters. In other cases, colony aggregation on particles was a strategy to fend off exploiters. Beyond these direct competitive mechanisms, we also uncovered how metabolic competition shapes community dynamics. Especially crucial were the initial stages of particle degradation, where degraders face challenges associated with resource limitations and diffusive GlcNAc loss, hindering chitinase production that is essential for community growth. Exploiters exacerbate the situation by siphoning off GlcNAc, thereby delaying or even completely obstructing growth of the coculture. These results reveal sensitive dependences of community viability on initial conditions and early degradation dynamics as an underappreciated hallmark of resource competition.

## Results

### Pairwise Cocultures Reveal Species-Specific Interactions.

**Fig. 1.**
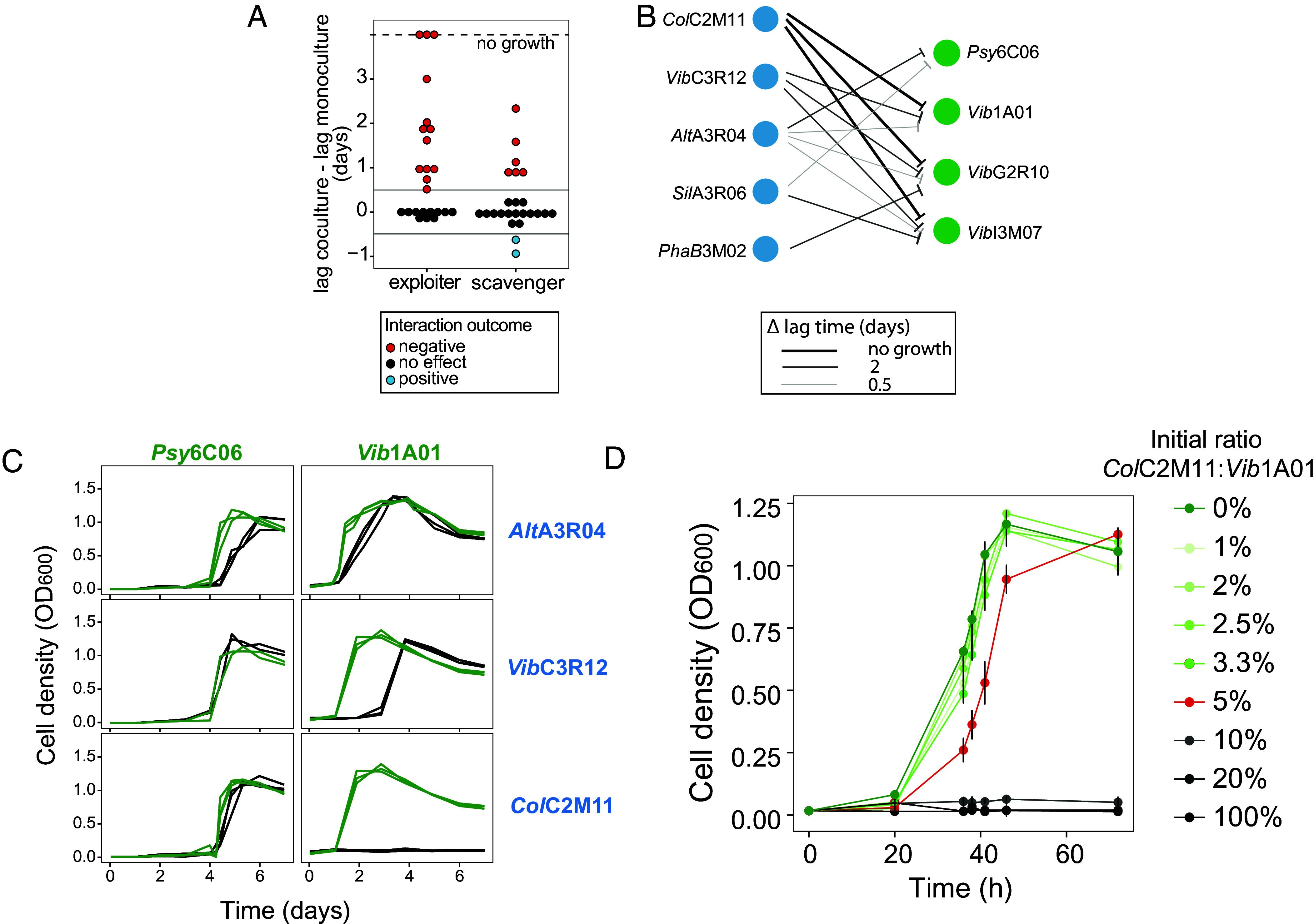
Pairwise cocultures show strain-specific effects on the outcome of chitin degradation. (*A*) Change in lag time (time to surpass OD_600_ 0.25), calculated by subtracting the lag time of degrader with either exploiter or scavenger cocultures from the lag time of monocultures. Red dots represent an inhibition phenotype, where exploiters or scavengers confer a relative increase in lag time greater than 12 h. Blue dots represent a positive interaction, where exploiters or scavengers decreases the lag time by more than 12 h. (*B*) Exploiter (blue)-induced lag times of degraders (green). Arrow width represents the increase in lag time in coculture. (*C*) Growth on colloidal chitin of selected degrader monocultures (green) and degrader-exploiter cocultures (black). The remaining pairwise coculture growth curves are in *SI Appendix*, Figs. S1 and S2. (*D*) Growth on colloidal chitin of *Vib*1A01 with *Col*C2M11 at different inoculation ratios, where the *Vib*1A01 density was 1 × 10^7^ cells/mL. The red curve highlights the critical inoculation ratio (*Col*C2M11:*Vib*1A01 = 0.05). Above the critical ratio cocultures grow (green lines) and below they do not (gray lines). Triplicate growth experiments are shown, where error bars are SD of the mean.

To identify strategies of how degraders and exploiters compete for GlcNAc as it is liberated from chitin, we used a model marine chitin-degrading community that consisted of bacteria previously categorized into three functional guilds based on their experimentally validated chitin degradation abilities (3): “degraders” express chitinases to release monomeric GlcNAc, “exploiters” do not express chitinases and depend on released GlcNAc for their growth, while “scavengers” cannot utilize GlcNAc or chitin and rely on other metabolites released by degraders or exploiters. We paired one of five exploiters or scavengers with one of four degraders (20 combinations each) and followed the growth of each coculture at 1:1 initial cell ratio (*SI Appendix*, Figs. S1 and S2, respectively). We hypothesized that exploiters would impart a higher frequency of inhibition due to competition for GlcNAc. The impact on degrader growth was quantified as changes in lag time, defined as the time required for a culture to surpass a total OD_600_ of 0.25 (*SI Appendix*, Table S3). Increased lag indicates inhibitory interaction while reduced lag indicates positive interaction. Out of the 40 cocultures studied, 19 showed inhibitory interactions (mostly increased lag times of at least 1 d), and only two exhibited positive interactions (Fig. 1*A*). Most inhibitory interactions (13 out of 19) were caused by exploiters, consistent with the hypothesis that many of these phenotypes are due to competition for GlcNAc. The six cases of degrader inhibition through scavengers (*SI Appendix*, Fig. S2) suggest that inhibition for reasons beyond GlcNAc competition can also occur.

In this work, we focus on the interactions between degraders and exploiters (Fig. 1*B*) due to the possibility of metabolic competition for GlcNAc. Only *Alt*A3R04 moderately inhibited the growth of all degraders (Fig. 1 *A* and *B* and *SI Appendix*, Fig. S1), while the other four exploiters inhibited some degraders but not others (*SI Appendix*, Fig. S1). For instance, while *Col*C2M11 and *Vib*C3R12 did not inhibit *Psy*6C06, they strongly inhibited *Vib1A01.* This suggests that species-specific inhibition is influenced by different competitive traits and may be condition dependent.

To discern cases of inhibition resulting from competition for GlcNAc rather than other factors, we will focus on the interactions between the three most inhibitory exploiters—*Co*lC2M11, *Vib*C3R12, and *Alt*A3R04—which together caused 10 of the 13 inhibitory interactions (Fig. 1*B* and *SI Appendix*, Fig. S1), and two degraders: the emerging model chitin degrader *Vib*1A01 (3, 45–47) and *Psy*6C06 (Fig. 1*C*). We note that *Col*C2M11 was the only exploiter capable of completely inhibiting degrader growth (thick arrows in Fig. 1*B* The extent of inhibition was dependent on the initial inoculation density. For example, the complete inhibitory effect of *Col*C2M11 on *Vib*1A01 vanished when *Col*C2M11 was seeded at densities 20-fold or lower (i.e., 5x10^5^ cells/mL in the red curve in Fig. 1*D* compared to 10^7^ cells/mL as initially tested in Fig. 1*C*). An influence of degrader preculture conditions was ruled out by inoculating cocultures with *Vib*1A01 grown in rich and chitin minimal medium, demonstrating nearly identical inhibition in both cases (*SI Appendix*, Fig. S3). Generally, whether the *Vib*1A01:*Col*C2M11 coculture grows or not seems to sensitively depend on the ratio of the initial inoculants (*SI Appendix*, Fig. S4), as the addition of 5% of exploiter (i.e., *Col*C2M11: *Vib*1A01 = 0.05:1) still resulted in growth (red curve), albeit with a delay, while the addition of 10% (i.e., *Col*C2M11: *Vib*1A01 = 0.1:1) completely inhibited the coculture (gray curve).

### Growth Inhibition by Toxin Secretion.

Similar inhibitory phenotypes identified through growth delays or arrests (Fig. 1*C* and *SI Appendix*, Fig. S1) may arise from different interaction mechanisms. To separate instances where the inhibition was not caused by competition for GlcNAc, we tested whether inhibition was due to secreted toxins or proteins. For this purpose, we examined whether cell-free supernatants from degrader:exploiter cocultures can inhibit degraders. Only the supernatant of the *Psy*6C06:*Alt*3R04 coculture inhibited the degrader monoculture, and the presence of a β-lactam antibiotic precursor in the coculture supernatant strongly suggested that this inhibition was due to antibiotics (*SI Appendix*, Supplemental Note 1). In contrast, as detailed in Supplemental Note 1, we found no evidence that *Col*C2M11 or *Vib*C3R12 secreted inhibitory compounds affecting *Vib*1A01.

### GlcNAc Uptake Kinetics and Metabolic Competition.

As the strong inhibitory effects of either exploiter *Col*C2M11 or *Vib*C3R12 do not involve toxin secretion, we next examined the ability of these two exploiters to compete with the degraders for GlcNAc monomers, focusing on why *Psy*6C06 is hardly affected by these two exploiters while *Vib*1A01 is very strongly affected (Fig. 1*C*). A simple hypothesis is that the exploiters have uptake kinetics that allows them to outcompete *Vib*1A01 but not *Psy*6C06 at low concentrations. This is particularly relevant in the initial phase of particle degradation, where the GlcNAc concentration is expected to be very low due to the low initial flux of its generation.

**Fig. 2.**
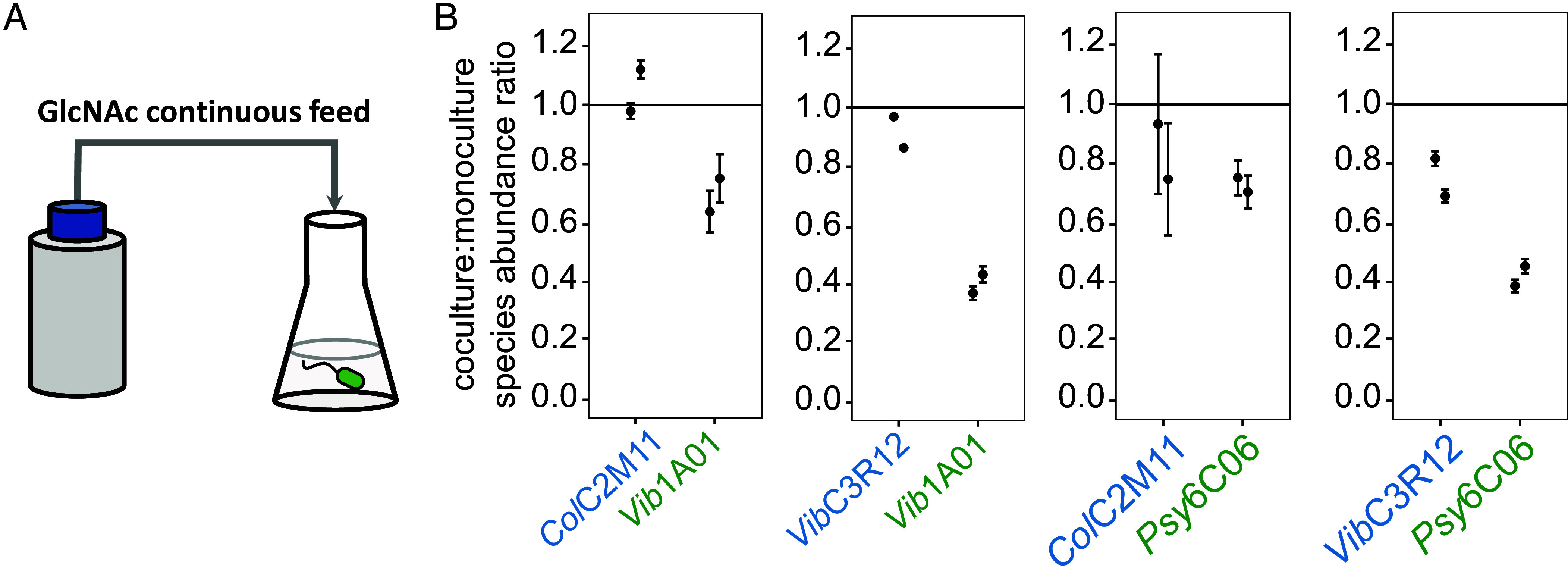
GlcNAc-limited fed-batch cocultures reveal similar uptake kinetics for degraders and exploiters. (*A*) Schematic of a GlcNAc-limited fed-batch reactor. GlcNAc is continuously dripped into a culture of the inoculated species that initially contains no carbon source. (*B*) Relative species abundance of degrader (green) and exploiter (blue) in GlcNAc-limited fed-batch cocultures compared to monoculture. Each species was inoculated at 1 × 10^7^ cells/mL and species abundance was determined after 72 h. Horizontal black lines are a visual guide corresponding to a ratio of 1, indicating no change. Error bars represent the SD of two technical qPCR replicates measurements, shown for each of two independent biological fed-batch cultures.

To quantify the relative uptake kinetics at low GlcNAc concentrations where direct measurement of growth rate is difficult, we employed a fed-batch reactor (48). We first competed *Vib*1A01 with each of the two exploiters (*Col*C2M11 and *Vib*C3R12) in fed-batch cultures. Bioreactor cultures were inoculated at 10^7^ cells/mL for each species, with the only carbon source being the continuously supplied GlcNAc to mimic gradual GlcNAc release during chitin hydrolysis by the degrader (Fig. 2*A*). The rate of GlcNAc feeding (15 µM/h initially and 7.5 µM/h toward the end of the 72 h growth period) was adjusted to mimic the rate of GlcNAc formation observed in bulk *Vib*1A01 monocultures between 0 and 24 h [~24 µM/h at OD_600_ 0.02 (38)]. Under such nutrient limitation, species specialized to consume scarce nutrients are expected to outcompete others. We assessed the abundance of each species in the coculture after 72 h by qPCR, normalizing species abundance in the coculture to the abundance of the corresponding monoculture in the fed-batch reactor with the same GlcNAc feeding rate and the same inoculation (Fig. 2*B*). Instead of the postulated complete dominance of exploiters over the degrader *Vib*1A01, we find that the abundance of *Vib*1A01 dropped only moderately, to about two thirds of that of *Col*C2M11 and about half of that of *Vib*C3R12 after 72 h of growth. Similarly, there was only a modest abundance drop of the degrader *Psy*6C06 in fed-batch cultures with either *Col*C2M11 or *Vib*C3R12 (Fig. 2*B*), suggesting that both degraders and the two exploiters possess similar uptake characteristics.

We used the abundance data to calculate growth rates of *Vib*1A01 and *Col*C2M11 in fed-batch coculture to estimate relative uptake kinetics under low GlcNAc conditions (*SI Appendix*, Supplemental Note 2). The less than twofold reduction in *Vib*1A01 abundance found after 72 h of competition leads to only ~20% difference in the growth rate of the competing species in the low nutrient regime. This moderate difference (*SI Appendix*, Fig. S10) does not come close to accounting for the strong inhibitory effects exerted by the exploiters on *Vib*1A01 (Fig. 1*C*), particularly, the complete inhibition of coculture growth when 10% (but not 5%) of *Col*C2M11 is added to the initial inoculant of *Vib*1A01 (Fig. 1*D*). Thus, the inhibition of *Vib*1A01 by *Col*C2M11 and *Vib*C3R12 cannot be explained by differences in uptake kinetics, indicating the presence of another mechanism.

### Role of Spatial Structure in Competitive Dynamics.

**Fig. 3.**
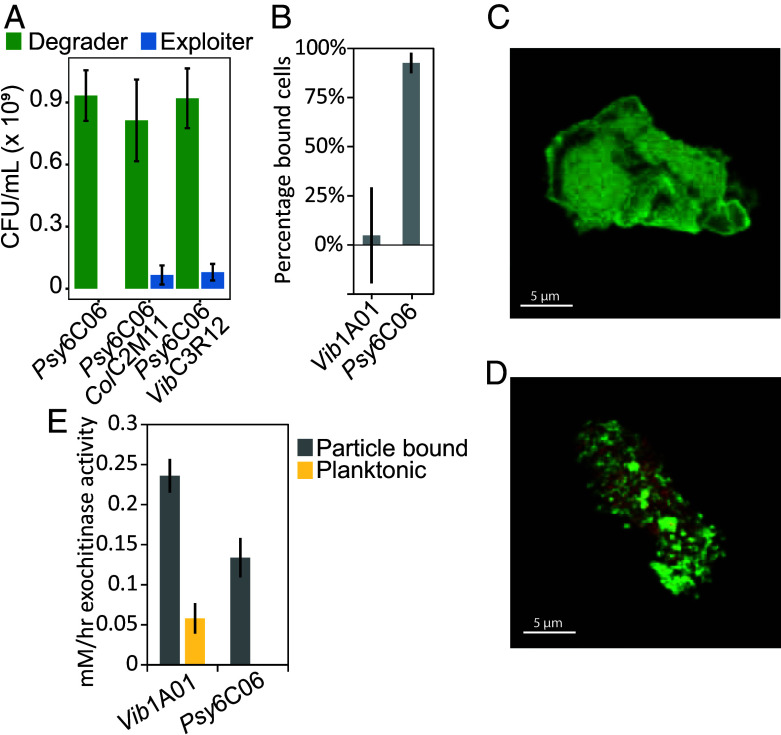
Strong particle binding increases degrader resilience to GlcNAc competition. (*A*) Early stationary phase colony-forming units of *Psy*6C06 (green) and exploiters (blue) in mono- or cocultures on colloidal chitin. (*B*) Percentage of cells bound to colloidal chitin 6 h after inoculation of monocultures, as determined by qPCR. (*C* and *D*) Microscope images of (*C*) *Vib*1A01 and (*D*) *Psy*6C06 growing on colloidal chitin. Red are the cells; green is the chitin. (*E*) Exochitinase activity in the planktonic (yellow) and chitin-bound (gray) fractions of each degrader after 24 h of growth on colloidal chitin. In all panels, error bars represent SD of the mean from triplicate experiments and triplicate qPCR measurements.

Despite the similarity in GlcNAc uptake characteristic, *Col*C2M11 and *Vib*C3R12 prevented or delayed the growth of *Vib*1A01 but not that of *Psy*6C06 (Fig. 1*C*). To confirm that *Psy*6C06 was indeed not affected by the exploiters, the final abundances of each species were determined after complete chitin degradation. Indeed, *Psy*6C06’s abundance was comparable in both mono- and cocultures (Fig. 3*A*).

One possible explanation for *Psy*6C06’s resilience to the exploiters is its ability to bind to the chitin particle surface (17). This binding allows *Psy*6C06 to privatize its chitinases even though they are extracellularly secreted by localizing its population to regions where chitinase and GlcNAc concentrations are highest. To test this hypothesis, we determined the fraction of each degrader that binds to colloidal chitin after 6 h. *Psy*6C06 was almost completely bound, while *Vib*1A01 showed no significant binding (Fig. 3*B*). This is consistent with a previous study which found a minor replicating fraction of *Vib*1A01 on the particle surface, with a larger planktonic and nondividing population (38). Using microscopy, we tested the binding of *Vib*1A01 and *Psy*6C06 on the particle surface during logarithmic growth of the monoculture. As expected, our images show dense colonies of *Psy*6C06 bound to the chitin surface, unlike *Vib*1A01 which rather appear to be dispersedly bound across the particle surface (Fig. 3 *C* and *D* and Movies S1 and S2). We further measured exochitinase activity on the particle and in the planktonic phase for both degrader monocultures at 24 h (Fig. 3*E*). In both cases the activity was higher on the particle surface, consistent with previous findings that *Vib*1A01 attaches chitinases to chitin particles (38).

To build on these results and gauge whether *Psy*6C06’s tight particle binding affects exploiter localization, we measured the fraction of chitin-bound versus planktonic cells in exploiter *Vib*C2M11 cocultures with either degrader at the last sampled timepoint before an OD_600_ increase was detectable (24 h for *Vib*1A01 and 72 h for *Psy*6C06 cocultures). Consistent with Fig. 3*B*, *Psy*6C06 was almost entirely particle-bound, whereas only ~10% of *Vib*1A01 was bound (*SI Appendix*, Fig. S6). In coculture with *Vib*1A01, *Col*C2M11 was nearly 100% particle-bound, but in coculture with *Psy*6C06, one third was in the planktonic phase. These results suggest that *Psy*6C06 gains its competitive advantage by aggregating on the chitin surface, and, to some extent, even preventing exploiters from occupying this niche. This aggregation allows *Psy*6C06 to privatize its chitinases and localize itself to the site of GlcNAc liberation, thereby increasing its access to resources and potentially enhancing its competitiveness in the coculture. Our data also indicate that tight particle binding enables *Psy*6C06 to limit exploiter access to the particle surface, providing an additional competitive advantage. In contrast, the inability of *Vib*1A01 to exclude exploiters on chitin particles may be a key vulnerability that allows it to be outcompeted when enough exploiters are present initially.

### Spatial Proximity but Not Direct Contact Mediates Degrader Inhibition.

**Fig. 4.**
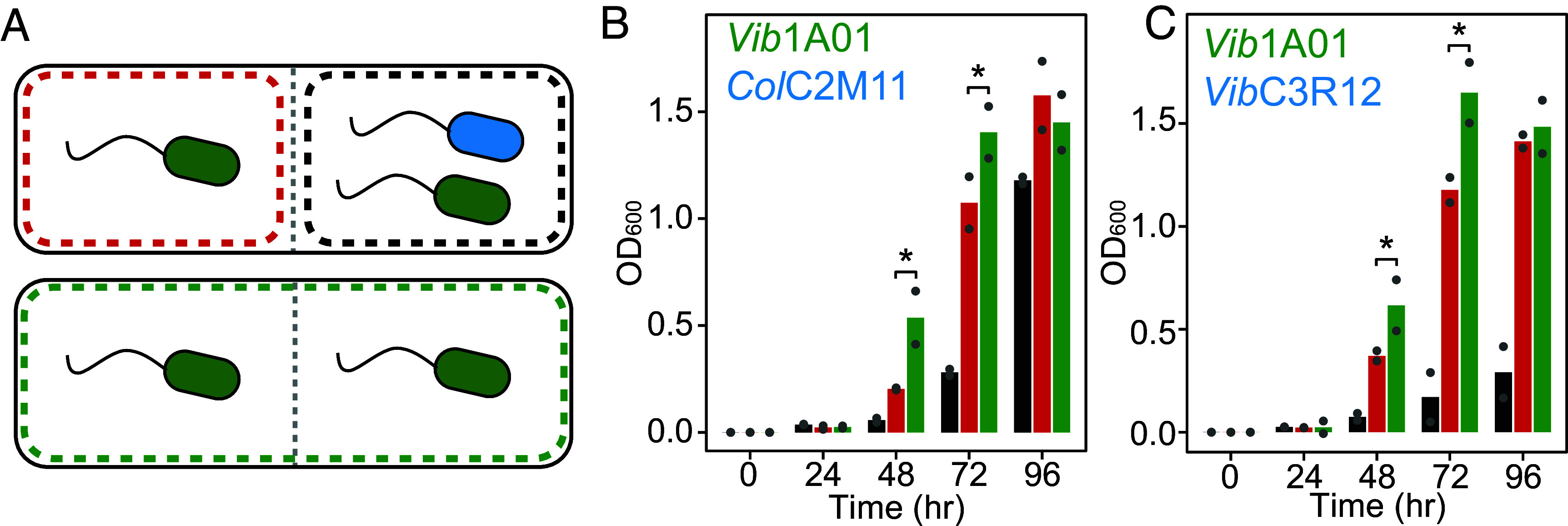
Exploiters inhibit degraders across a membrane without direct contact. (*A*) Setup of membrane-separated coculture chambers with the degrader *Vib1A01* (green) and one of the two exploiters, *ColC2M11* or *VibC3R12* (blue). (*B*) Growth in membrane-separated growth chambers: *Vib1A01* monocultures on both sides (green bars) and *Vib1A01* monoculture (red bars) separated from *Vib1A01*:*ColC2M11* coculture (black bars). (*C*) Same as (*B*) but with the exploiter *VibC3R12*. Coculture experiments were performed in duplicate, cells were inoculated at 1 × 10^7^ cells/mL. Stars represent significant changes (*P* < 0.05, one-tailed *t* test) when comparing the *Vib1A01* monocultures with or without the exploiter inoculated on the other side of the membrane (i.e., comparison of red and green bars only).

Next, we investigated the cause of the hypersensitivity of degrader *Vib*1A01 to the two exploiters despite their metabolic similarity. We first examined the role of spatial proximity on the inhibition of *Vib*1A01. Since *Vib*1A01 cannot exclude *Col*C2M11 and *Vib*C3R12 from the particle surface (Fig. 3*C*), we tested whether direct contact was a requirement of the inhibitory dynamics in these cases. To do this, we used a device that separated two culture chambers with a 0.1 µm pore size membrane, allowing the passage of metabolites or secreted toxins, but not of cells (49). With colloidal chitin as a sole carbon source, we inoculated *Vib*1A01 in both membrane-separated chambers and the exploiter in only one chamber (top cartoon, Fig. 4*A*). The distance dependence of inhibitory effects resulting from the coculture would be apparent when comparing growth of *Vib*1A01 in this device with that in another device containing only *Vib*1A01 in both chambers (bottom cartoon, Fig. 4*A*). Interactions requiring the colocalization of the two species would exclusively occur in the coculture chamber (black box in top cartoon, Fig. 4*A*) while interactions mediated by diffusible molecules would only be visible in the monoculture chamber (red box in top cartoon, Fig. 4*A*).

We find statistically significant inhibition of *Vib*1A01 by *Col*C2M11 and *Vib*C3R12 to occur in the adjacent degrader-only chamber (compare the red and green bars, Fig. 4 *B* and *C*), suggesting the involvement of small molecules diffusing across the membrane separating the cultures. This allows us to rule out inhibitory processes that depend exclusively on cell-to-cell contact. On the other hand, physical proximity clearly plays an important role since there are drastic differences between the black and red bars (Fig. 4 *B* and *C*); this suggests a quantitative dependence of the inhibitory effect on the separation distance. Since cell-free supernatant did not have an inhibitory effect (*SI Appendix*, Supplemental Note 1), it is likely that the small molecules mediating the distance-dependent inhibition could be monomers or oligomers of GlcNAc. These results point to the likely role of metabolic competition mediating interaction between *Vib*1A01 and the exploiters.

### Sensitivity of Chitin Culture to Small Changes in GlcNAc and Chitinases.

**Fig. 5.**
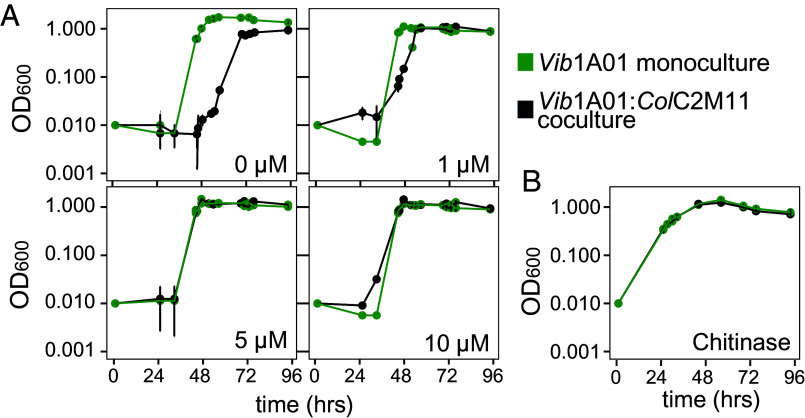
Small increases in initial GlcNAc or chitinase concentrations eliminate exploiter-induced growth delays. Growth of *Vib*1A01 monoculture (green) and coculture (black) with *Col*C2M11 with variation in initially supplied (*A*) nongrowth supporting concentrations of GlcNAc or (*B*) purified chitinase. Error bars represent SD of the mean from triplicate cultures.

The sensitivity of cocultures to initial inoculum density point to an important role of initial conditions in determining coculture outcome. To test whether initial nutrient conditions indeed influence coculture dynamics, we supplemented the cultures with low concentrations of GlcNAc (1 to 5 µM). This supplementation restored the monoculture growth phenotype, demonstrating that inhibition depends critically on nutrient availability at the onset of colonization (Fig. 5*A*). Importantly, this result is consistent with the observation that *Vib*1A01 and the exploiters have comparable GlcNAc uptake affinities; otherwise, *Vib*1A01 would be completely outcompeted at such low concentrations and growth would not occur.

Although the addition of 1 to 5 µM GlcNAc can at most support a 2 to 10% increase in *Vib*1A01 biomass, the effect of such a small shift is likely amplified because chitinases and accessory proteins constitute roughly one third of the particle-associated *Vib*1A01 proteome (38). A slight increase in initial GlcNAc uptake may therefore result in higher chitinase concentrations on the particles, boosting particle degradation. Supporting this, direct supplementation of cocultures with purified chitinases also abolished inhibition and eliminated lag entirely (Fig. 5*B*). Together, these data suggest that the increase in lag exerted by the exploiters have to do with them siphoning away small amounts of GlcNAc from the degraders during the initial stages of coculture growth. These low initial concentrations are crucial since they would have otherwise contributed to chitinase synthesis, resulting in sustained GlcNAc release and particle degradation.

### A Model of Metabolic Competition Recapitulates Coculture Sensitivity to Initial Conditions.

**Fig. 6.**
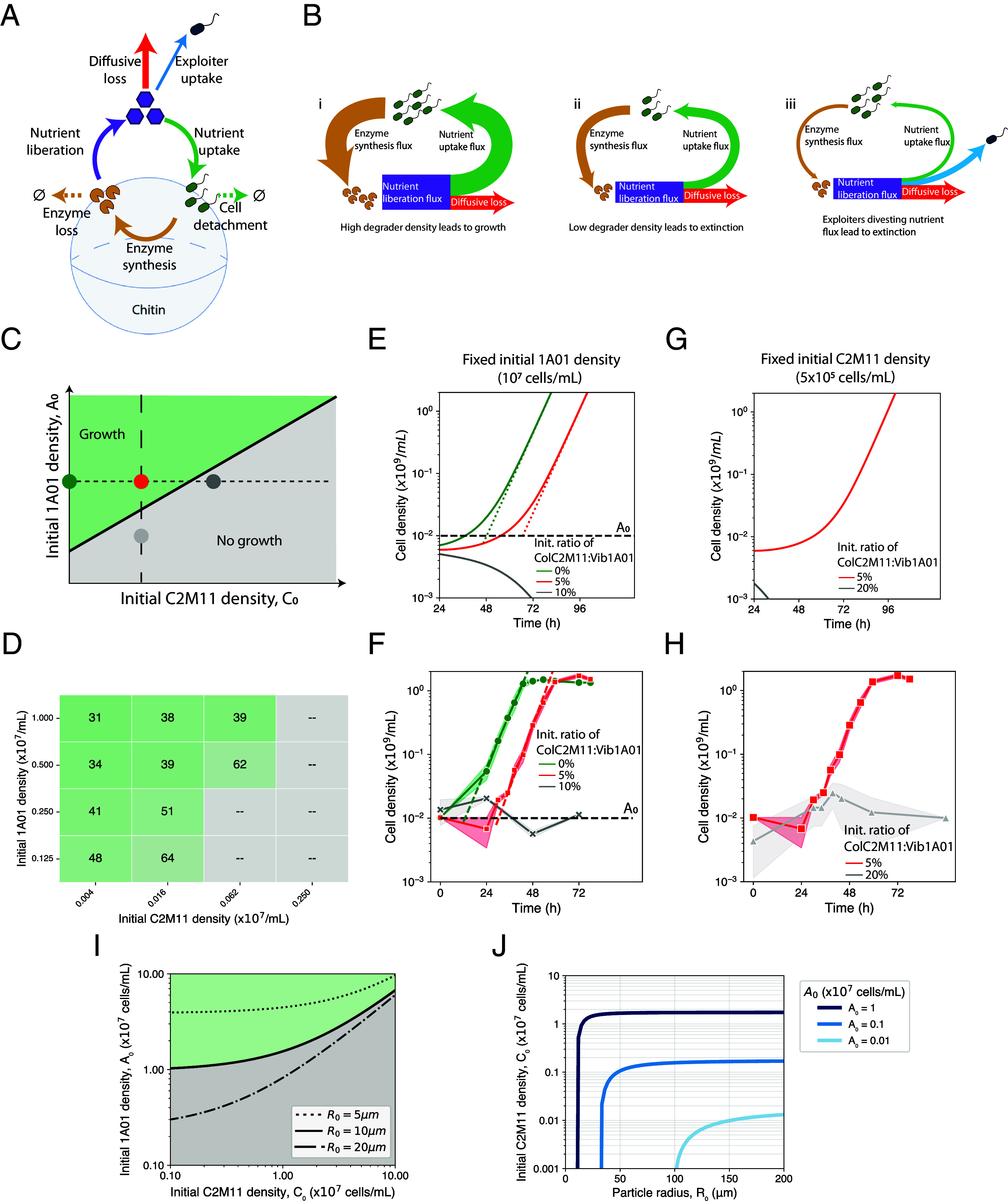
A metabolic model recapitulates a phase transition in the growth of the chitin coculture. (*A*) Model of chitin-degrader dynamics. Degraders (green cells) synthesize chitinases (yellow pacmans), which in turn generate a GlcNAc liberation flux (purple arrow). The GlcNac molecules (purple hexagons) are taken up by the degraders (green arrow), the exploiters (blue arrow), and leak away due to diffusion (red arrow). Degraders detach from cells (dashed green arrow) and enzymes turn over (dashed yellow arrow) as empirically determined by Guessous et al. (38). Most parameters in this simple model are experimentally constrained as described in *SI Appendix*, Supplementary Note 3. (*B*) Cartoon depicting the partition of the liberated GlcNAc flux in different scenarios: i) when enough degraders and chitinases are present initially, the GlcNAc liberation flux is large (thick purple box), and cell growth (green arrow) and chitinase production (yellow arrow) is hardly affected by diffusive leakage (red arrow), leading to exponential growth. ii) At low initial degrader densities/chitinase concentration, the GlcNAc liberation flux is reduced (thin purple box), and most of the GlcNAc liberated is lost to diffusion; this drastically reduces the GlcNAc available for uptake, thus reducing cell growth (as indicated by the thinner green and yellow arrows). iii) Exploiters can enhance the effect in (ii) by siphoning GlcNAc away (blue arrow) from the degraders and hence from chitinase production, leading to further growth reduction (very thin green and yellow arrows). (*C*) An illustrative phase diagram in the space of the initial degrader and exploiter densities. The black line separates the growth and no-growth phases. The green circle represents a growing monoculture. The red circle indicates 5% exploiter addition, which pushes the system closer to the phase transition. The gray circle indicates 10% exploiter addition, which tips the system to the no-growth phase. (*D*) Lag times for chitin coculture growing on various initial degrader and exploiter densities; a snapshot of the coculture OD at 24 h after seeding is shown in *SI Appendix*, Fig. S4. Lag time is extracted from coculture growth curves as defined above (OD_600_ > 0.25). Gray entries correspond to cocultures that did not grow after ~100 h. (*E*) Dynamics of the culture according to numerical solutions of the model illustrated in panel *A*; see *SI Appendix*, Supplementary Note 3 for model details and parameters used. The green, red, and gray lines represent the monoculture, and coculture with 5% and 10% initial exploiters, respectively, with the initial degrader at 10^7^ cells/ml (horizontal dotted line, panel *C*). The monoculture grows exponentially after some lag, with the growth rate (slope of the dashed lines) being 0.16/h. The addition of 5% exploiters (red curve) grows at the same rate, but with the lag increased by ~20 h. The addition of 10% exploiters completely inhibited growth (gray line). (*F*) A detailed view of the growth curve for the chitin monoculture of *Vib1A01* (green) and coculture with *ColC2M11* (red), with initial cell density being 10^7^ cells/mL and 5 × 10^5^ cells/mL, respectively. Gray symbols represent the nongrowing coculture with initial density of *Col*C2M11 being 10^6^ cells/mL (10% *Vib*1A01). The shading represents the SD from three different biological replicates. The population growth rate is 0.18/h for the monoculture and 0.16/h for the coculture with 5% *Col*C2M11, with the latter having a ~18 h longer lag. The effect of the exploiters on lag produced by the model is in good agreement with the experiment; but the lag of the monoculture is overestimated in the model due to simplifying approximation made; see *SI Appendix*, Supplementary Note 3 for discussion. (*G*) Dynamics of the cocultures according to the numerical solution of the model, with the initial density of *Col*C2M11 fixed at 5 × 10^5^ cells/mL, and with varying initial density of *Vib*1A01 (vertical dashed line in panel *C*): 10^7^ cells/mL (red, same as the red line in panel *E*), and 2.5 × 10^6^ cells/mL (gray). The inhibition caused by the exploiter (gray line) is alleviated by higher initial degrader density. (*H*) Experimental growth curves of the cocultures with the same initial conditions as in *G*. The experimental results are in good agreement with the numerical solution, wherein the gray line exhibits no growth, while the red line shows growth (same as red line in panel *F*). (*I*) Quantitative phase diagram of the initial densities of the degrader (A_0_, y-axis) and exploiter (C_0_, x-axis) based on simulating the dynamics of the system as described in *SI Appendix*, Supplementary Note 3 (Eq. N3.3.4 and N3.3.5). Note the qualitative similarity with panel *C*. The solid black line represents the phase boundary between growth (green area) and no growth (gray area) for $R_{0}=10\;\mu m$. Our model predicts that for smaller particles (dotted line, $R_{0}=5\;\mu m$) the extinction phase is extended, since the effect of nutrient diffusion is enhanced. Conversely larger particles (dashed-dotted line, $R_{0}=20\;\mu m$) have an extended growth phase. (*J*) For various fixed initial degrader densities ($A_{0}$, different shades of blue), we show the critical exploiter density above which growth no longer occurs.

The ability of degraders to establish growing cultures on chitin stems from the positive feedback between chitinase production by the degrader and its uptake of the liberated GlcNAc, that in turn fuels further chitinase synthesis. Qualitatively, an inhibitory effect of exploiters on degraders can arise from breaking this positive feedback: by siphoning GlcNAc away from the degraders, the exploiters can undermine the degraders’ ability to synthesize chitinases, preventing particle degradation altogether. However, the puzzling aspect of the data presented above is that the observed inhibitory interaction is very sensitive to the initial exploiter density (Fig. 1*D*). Specifically, cocultures starting with 5% of *Col*C2M11 showed little inhibitory effect (manifested by a moderate lag time), but those starting with 10% of *Col*C2M11 were unable to grow. The origin of this strong sensitivity is unclear, especially given that the uptake efficiencies of *Vib*1A01 and that of the exploiters are comparable (Fig. 2*B* and *SI Appendix*, Supplemental Note 2). The conceptual model shown in Fig. 6 *A* and *B* identified several key ingredients linked to the observed inhibition of *Vib*1A01 by *Col*C2M11 and *Vib*C3R11: metabolic competition for GlcNAc in the early phase of the coculture, involving spatial proximity of the degrader and the exploiter. Here, we construct a metabolic model with minimal ingredients that recapitulates the observed strong sensitivity to inhibition by exploiters.

To be sensitive to small changes in the initial exploiter density, we hypothesize that the degrader-chitin system is poised close to a phase transition where small changes in the initial GlcNAc flux could substantially affect degrader growth. Generally, a strong nonlinearity is needed to amplify small perturbations such as those caused by the presence of exploiters. However, the simple nutrient dynamics depicted in Fig. 6*A*, comprising of Monod growth (green arrow), chitinase synthesis (brown arrow), and Michaelis–Menten enzyme kinetics (purple arrow), contain only linear or even sublinear dynamics and thus lack a source of amplifying nonlinearity.

Here, we point to a simple physical effect that provides an effective amplifying power to the inhibitory effect of the exploiters on the degraders – the leakage of the liberated GlcNAc away from the degraders by diffusion (red arrow, Fig. 6*A*). As described in detail in Supplemental Note 3, while the existence of such a diffusive flux is inevitable, it is particularly large for the small colloidal chitin particles studied here, due to the inverse square dependence on surface curvature. Numerically, we estimate that for particles of 10 µm radius (Fig. 4*C*), the GlcNAc flux leaked away by diffusion would be comparable to the uptake flux by *Vib*1A01 cells at a density of 2 × 10^7^ cells/mL. This indicates that a density of this order, which is on the order of the initial degrader density in our experiments, is the minimum density necessary to capture enough GlcNAc to get the culture growing (Fig. 6 *B*, *i* and *ii*). Our experimental system, which is poised near this initial critical density, is close to the borderline of a phase transition between growth and no growth. It is then plausible that a small reduction in the initial amount of GlcNAc captured by the degraders, resulting from the siphoning of GlcNAc by a small number of exploiters (blue arrow, Fig. 6 *A* and *B*, *iii*) could tip the system from growth to no-growth, as illustrated by the phase diagram in Fig. 6*C*. Conversely, a small increase in the initial amount of GlcNAc captured by degraders could ensure growth of the population (Fig. 5*A*). The structure of the growth/no-growth regions in the space of initial degrader and exploiter densities indicated in Fig. 6*C* is echoed qualitatively in Fig. 6*D* by the lag-time data extracted from growth curves collected across many combinations of initial inoculant densities (see *SI Appendix*, Fig. S4 for a snapshot).

To probe the transition between the two regions more closely, we performed a numerical calculation of the model depicted in Fig. 6*A*; see *SI Appendix*, Supplementary Note 3. The model qualitatively reproduced the basic phenomenon of coculture sensitivity to small changes in the initial exploiter density (Fig. 6*E*). The addition of 5% of exploiters lengthens the monoculture lag by about 20 h (compare the red and green curves), and the addition of 10% of exploiters completely inhibits growth (gray curve), matching the experimental observation (Figs. 1*D* and 6*F*). In addition to exploring the transition for increasing initial density of *Col*C2M11 at fixed initial density of *Vib*1A01 (horizontal dotted line, Fig. 6*C*), we also probed the transition in the orthogonal direction by changing the initial density of *Vib*1A01 while fixing the initial density of *Col*C2M11 (vertical dashed line, Fig. 6*C*). The same kind of transition is obtained, numerically and experimentally (Fig. 6 *G* and *H*, respectively). We note that most parameters used in the numerical calculation are constrained by measurements in this work and from Guessous et al. (38) for the degrader *Vib*1A01 (listed within Supplementary Note 3), with one experimentally inaccessible parameter (the initial amount of chitinase synthesized by *Vib*1A01 before substantial GlcNAc intake) chosen to place the growth transition at the observed location; see *SI Appendix*, Supplemental Note 3. Importantly, the three key ingredients deemed necessary for the occurrence of the abrupt growth transition, the aforementioned diffusive leakage flux, and the turnover of degraders and chitinases on particles, have all been established experimentally for *Vib*1A01 (38). This model quantitatively supports the hypothesis that siphoning of GlcNAc by exploiters during the initial stages can stall particle degradation altogether and reveals a very sensitive phase transition.

## Discussion

Here, we present molecular and physiological evidence demonstrating how different competition strategies for the chitin monomer GlcNAc contribute to setting the outcome of polysaccharide degradation. Pairwise coculture experiments revealed widespread species-specific inhibition of degraders through exploiters that were brought about by distinct mechanisms. For instance, the exploiter *Alt*A3R04 secreted a β-lactam antibiotic to inhibit the growth of the degrader *Psy*6C06. On the other hand, the degrader *Psy*6C06 evaded exploiter competition by aggregating on chitin particles, thus privatizing its chitinases and the resulting degradation products, which in turn enhances per capita growth rates (17) and partially excludes exploiters from the particle surface.

In several cases, exploiters can completely suppress coculture growth, to the detriment of the exploiters themselves. The interaction dynamics between the degrader *Vib*1A01 and the exploiters *Col*C2M11 and *Vib*C3R12 highlight a fine balance between GlcNAc liberation rates during initial particle degradation, its leakage due to diffusion, and its partitioning between competitors. This interaction, which is characterized in detail for the degrader *Vib*1A01 and the exploiter *Col*C2M11, is highlighted by a very sensitive dependence of coculture growth on the initial exploiter density: an addition of 5% of exploiters hardly affected the coculture while 10% of exploiters completely suppressed growth.

This sensitivity of the system to exploiter density can be attributed to the compounding effect of a small flux of GlcNAc liberation by an initially low density of the degrader *Vib*1A01 (which detach readily from chitin particles (38)) as well as substantial loss of GlcNAc to diffusion away from particles, especially for small particles. Exploiters that compete for GlcNAc would further exacerbate GlcNAc limitations caused by diffusive loss, leading to a severe growth suppression by a low density of exploiters (*SI Appendix*, Supplemental Note 3). As shown in Fig. 6 *C*–*H*, the growth transition driven by changes in the initial density of exploiters is the same as the transition driven by changes in the initial density of degraders. The latter can be viewed as a manifestation of the Allee effect, that the growth of a population (the degraders) requires a critical initial density (17, 50, 51) (*SI Appendix*, Fig. S15). In this light, metabolic competition can be viewed as a mechanism that increases the critical density of the Allee transition (Fig. 6*C*): the suppression of coculture growth by exploiters can be viewed as the result of the initial density of degraders falling below the now-increased critical density necessary for growth.

While both density-dependent growth effects (52) and the deleterious impact of cheaters on population fitness (52, 53) have been documented previously, prior work has largely examined these effects independently or in single-species systems with isogenic exploiters unable to secrete public-goods generating enzymes, which are discrete mutations of the ancestor (54). As a result, both the cheater and the degrader have the same physiological behavior and phenotype, which is not necessarily the case for isolates in wild and diverse populations. Moreover, our study couples these processes to a spatially structured environment that reflects the more complex ecological reality of chitin degradation. Despite these complexities, we were able to identify two pairs of strains with similar physiologies when grown on labile carbon substrates. For these pairs, the outcome of the multispecies degrader:exploiter cocultures on chitin recapitulated similar density-dependent behaviors as those found in engineered single-species systems (54). Previous models of chitin degradation, such as detailed agent-based simulations (17), have also resulted in threshold behavior in the initial cell density. However, these typically require fine-tuning many loosely constrained parameters and do not provide a simple, experimentally grounded mechanism for the sensitivity observed. Our model quantitatively predicts the absolute critical inoculum density for a given particle size and exploiter load (Fig. 6 *I* and *J*), given a particular bacterial strategy (e.g., detachment-dominated dynamics for *Vib*1A01 or enzyme privatization for *Psy*6C06), providing causal factors underlying measurable ecological traits. This analysis, which relies on model parameters that are experimentally constrained, identifies the key role of diffusion in setting the observed sensitivity to initial exploiter densities. The effect of diffusion is manifested by the strong dependence on particle size (Fig. 6 *I* and *J*).

Importantly, the outcomes of our quantitative analysis suggest that the inhibitory effect due to nutrient loss by diffusion and siphoning by exploiters are relevant to natural marine environments, where bacterial densities are typically 10- to 100-fold (55, 56) lower than in our experiments, and particles have a large size range (57). In Fig. 6*J*, we observe a step-like dependence of chitin degradation on particle sizes, with a minimal size for growth for different initial densities of *Vib1A01* even with vanishing *ColC2M11* densities (vertical rise of the lines). Competition by exploiters is seen to “round the corner” of the step-like feature, with the rounding becoming more prominent at lower initial degrader densities. In the parameter range of central relevance to the ocean (light blue curve), Fig. 6*J* predicts that the outcome of chitin degradation even for particles as large as ∼100 μm is easily affected by the presence of exploiters. This effect would thus act to decelerate particulate organic carbon turnover down the water column and contribute to the long tail of the Martin curve (referring to the widely used empirical relationship between particulate carbon flux and ocean depth) (58), ultimately constraining the remineralization potential of marine microbes and its effect on global carbon cycling (59).

Our study complements previous work on metabolite partitioning by highlighting the temporal dimension of species-specific interactions and competition. Many cross-feeding studies treat metabolite partitioning as a static snapshot (1, 3, 5, 6, 60). The focus is usually on which compounds a species consumes and how this defines competitive interactions. Our results expand on these studies by showing that snapshot-based metabolite partitioning occurs, but its impact is often overridden by other competitive strategies and by environmental contexts that shift during community assembly.

Our work also shows that similar phenotypes, such as delayed growth, can arise from distinct underlying mechanisms, depending on the organisms involved and their ecological context. Within our chitin-degrading community, these strategies include toxin secretion, particle aggregation, and GlcNAc siphoning. The implications of our findings extend beyond the specific cases studied here, suggesting parallels in other polysaccharide-degrading microbial communities that are similarly influenced by resource competition (3, 8, 11, 60–64). Notably, our results show that microbial community behavior is shaped by more than the inherent traits of organisms; competitive strategies are finely tuned to specific ecological contexts. A key feature of the growth transition observed in this study is that variability in initial colonization conditions sensitively modulates interaction outcomes. Even minor changes in population densities or nutrient levels can lead to drastically different consequences. This sensitivity has profound implications for laboratory experiments, potential clinical interventions, as well as for natural environments, where slight variations in conditions can produce drastically different results for the community.

## Methods

### Materials and Chemicals.

All chemicals were obtained from Sigma-Aldrich unless noted otherwise. Media used are Marine Broth 2216 (Thermo Fisher Scientific, Difco, no. 279100) or MBL minimal medium.

MBL contains 1 mM phosphate dibasic, 1 mM sodium sulfate, and 50 mM HEPES (pH 8.2), and three additional diluted stocks: First, fourfold concentrated seawater salts (NaCl, 80 g/L; MgCl_2_*6H_2_O, 12 g/L; CaCl_2_*2H_2_O, 0.6 g/L; KCl, 2 g/L). Second, 1,000-fold concentrated trace minerals (FeSO_4_*7H_2_O, 2.1 g/L; H3BO3, 30 mg/L; MnCl_2_*4H_2_O, 100 mg/L; CoCl_2_*6H_2_O, 190 mg/L; NiCl_2_*6H_2_O, 24 mg/L; CuCl_2_*2H_2_O, 2 mg/L; ZnSO_4_*7H_2_O, 144 mg/L; Na_2_MoO_4_*2H_2_O, 36 mg/L; NaVO_3_, 25 mg/L; NaWO_4_*2H_2_O, 25 mg/L; Na_2_SeO_3_*5H_2_O, 6 mg/L, dissolved in 20 mM HCL). Third, 1,000-fold concentrated vitamins (riboflavin, 100 mg/L; d-biotin, 30 mg/L; thiamine hydrochloride, 100 mg/Liter; L-ascorbic acid, 100 mg/L; Ca d-pantothenate, 100 mg/L; folate, 100 mg/L; nicotinate, 100 mg/L; 4-aminobenzoic acid, 100 mg/L; pyridoxine HCl, 100 mg/L; lipoic acid, 100 mg/L; nicotinamide adenine dinucleotide (NAD), 100 mg/L; thiamin pyrophosphate, 100 mg/L; cyanocobalamin, 10 mg/L, dissolved in 10 mM MOPS pH 7.2).

### Preparation of Colloidal Chitin.

10 g of powdered chitin (Sigma-Aldrich, C7170) was dissolved in 100 mL of concentrated phosphoric acid (85% by weight) and then placed at 4 °C for 48 h. Roughly 500 mL of deionized water was added to this mixture and shaken vigorously until all chitin precipitated. The precipitate was filtered using regenerated cellulose paper (MACHEREY-NAGEL, MN615). Chitin precipitate was then placed in cellulose dialysis tubing (approximately 13 kDa, Sigma-Aldrich D9652-100FT) and dialyzed with fresh deionized water daily for 3 d to remove residual phosphoric acid and oligomers. Following dialysis, the pH was adjusted to 7 with 1 M NaOH and homogenized using Bosch SilentMixx Pro blender. The colloidal chitin was sterilized by autoclaving.

### Bacterial Strains and Culturing.

All bacterial species were stored in glycerol stocks at −80 °C. Before use, they were streaked onto Marine Broth 2216 plates with 1.5% agar (BD, no. 214010) and placed at room temperature until colonies formed. Overnight precultures were prepared by inoculating a single colony into 2 mL of Marine Broth 2216 and placed in a 27 °C shaker overnight.

Unless otherwise noted, growth experiments were performed as follows. The medium used is MBL. Cells were inoculated at a density of 1 × 10^7^ cells/mL, as calculated based on their optical densities (*SI Appendix*, Table S2). As a carbon source, chitin was supplied at 2 g/L. Cultures were grown in 24 deep well microtiter plates (Kuhner) with 3 mL volumes and shaken at 200 rpm at 27 °C. Growth was monitored with OD_600_ by harvesting 100 and 200 μL of culture for measurement in a Tecan Sunrise plate reader. To prevent suspended chitin from interfering with OD_600_ measurements, the 24 deep well plate was centrifuged for 30 s at 1,000 rcf and the optical density of the supernatant was measured.

### Testing for Toxicity in Cell-Free Supernatant.

A degrader:exploiter coculture or degrader monoculture was grown on 2 g/L MBL in 3 mL volumes in a 24 deep well plate in 6 replicates. As soon as the cultures entered into early stationary phase, the supernatant was harvested by centrifuging at 4,000 rcf for 5 min followed by filtration though a 0.22 μM membrane. Large molecules were removed from half of the supernatant using an Amicon Ultra 10 kDa cutoff filter before being sterilized again with a 0.22 μM filter. Both supernatants (with and without large molecules removed) were diluted 1:2 with fresh MBL containing 4 g/L chitin, inoculated with the degrader, and growth was measured over time.

### Determining Cell Abundance with qPCR.

Cell abundance in fed-batch reactors was measured using qPCR. Cell cultures were preprocessed as follows: 40 μL of culture was added to 10 μL of Chelex mastermix (25% wt Chelex 100, 200 to 400 mesh, and 100 mg/mL proteinase from *Aspergillus melleus* Sigma P4032-5G). This mixture was incubated at 56 °C for 60 min, and 95 °C for 10 min, and was further diluted 1:10 in nuclease-free water. qPCR was performed using Promega GoTaq qPCR mastermix in 15 μL reactions using 6 μL of the diluted Chelex reaction as a template. Readings were performed using a QuantStudio 3 (Thermo Scientific) qPCR machine. Genome-specific primers amplified a 75 to 150 bp region on the genome with a measured amplification efficiency of 80 to 105% (*SI Appendix*, Table S4). Cell abundances were determined from the qPCR cycle threshold (Ct) values by comparing Ct values to a standard curve of Ct values measured from samples with known CFU/mL concentrations. All qPCR measurements were performed with two technical replicates that were averaged.

### Chitin Binding Affinity Assay.

To determine the fraction of cells that binds to chitin in the first 6 h, species were inoculated into MBL containing 2 g/L colloidal chitin or no colloidal chitin, in four replicates each. After 6 h, 500 µL of culture was collected, centrifuged at 1,000 rcf for 30 s to separate suspended chitin and any bound cells from the medium. The supernatant was harvested, and the cell concentration was measured using qPCR.

### Measuring Species Abundance in Chitin Cocultures.

Cultures were grown on colloidal chitin until early stationary phase, when the OD_600_ does not rise for at least 2 h and all visible chitin has been consumed. Total CFU/mL are counted by plating the cultures onto MB2216 agar plates. The identity of 25 colonies derived from each culture were determined using qPCR, which is used to calculate the fraction of each species in the total CFU/mL.

### Exochitinase Assay on Particle Surfaces.

To compare exochitinase activity between the particle-bound and planktonic fraction, exochitinase activity was measured using an in vitro enzyme assay that relies on the enzymatic hydrolysis of 4-nitrophenyl N-acetyl-β-d glucosaminide to p-nitrophenol. *Vib*1A01 and *Psy*6C06 were grown on colloidal chitin for 24 h and 500 µL of sample was centrifuged at 1,000 rcf for 30 s. The supernatant was removed as the planktonic fraction. Fresh MBL was added back to the pellet, containing all bound cells and chitinases, to bring the volume back to 500 µL. A mastermix solution was prepared that contains 100 mM sodium acetate and 2 mM 4-nitrophenyl N-acetyl-β-d glucosaminide. 150 μL of this mastermix was added to 150 μL of the bound or planktonic fraction. The reaction was allowed to proceed for 30 min at room temperature. To quench the reaction, 100 μL of the enzyme reaction was centrifuged at max speed and the supernatant was added to 100 μL of 10% ammonium hydroxide, 2 mM EDTA. The assay provides a colorimetric readout at 405 nm. Pure p-nitrophenol is used as a standard to generate a calibration curve.

### Untargeted Mass Spectrometry.

Metabolomics was performed using Liquid Chromatography coupled to an Agilent 6520 Time of Flight Quadrupole Time of Flight Mass Spectrometer in positive mode, 4 GHz, high-resolution mode. The column was an Agilent EC-CN Poroshell column (50 × 2.1 mm, 2.7 µM), operated isocratically, which has been shown to reduce the interference of salts on metabolite ionization (65). The buffer contains 10% Acetonitrile (Chromasolv), 90% water, and 0.01% formic acid. The flow rate was 350 µL/min. The sample was diluted 20-fold in MillQ water prior to measurement, and 3 µL was injected every 2 min. Raw data for all measurements were subjected to a spectral processing and alignment pipeline using Matlab (The Mathworks, Natick) as described previously (66). Ions were annotated with a tolerance of 5 mDa against a curated compound library that contains metabolites predicted to be in at least one species used in this study based on the BioCyc database (67). Annotated metabolites and their intensities can be found in Dataset S1. All raw spectral files have been deposited to the MassIVE database (MassIVE MSV000093541, 10.25345/C5K931H62).

### Fed Batch Reactors.

Six parallel nutrient-limited fed batch reactors were set up as follows in a 25 °C controlled room. First, 20 ml of MBL medium with no carbon source was placed into a 250 mL Erlenmeyer flask (bioreactor) containing a magnetic stir bar and a silicone sponge closure to minimize contamination (Sigma, C1046). Feed medium was placed in a separate bottle containing a pierceable rubber cap (Fisher, 15896921). Here, the feed medium contains MBL supplemented with 1 mM GlcNAc. A feed tube was configured to transport medium between the feed bottle and the Erlenmeyer flasks, powered by a peristaltic pump (Ismatec IPC8). First, a stainless-steel needle (18 gauge, 6 inch stainless steel 304 syringe needle Sigma Z102717) withdraws medium from the feed bottle. This is connected to a PharMed BPT tubing (2.79 mm) through a male leur fitting for 1/8 in tubing (Sigma 21016). Next, this tube is connected to a PharMed 2-stop tubing 0.25 mm (Ismatec 95723-12) using another 1/8 in male leur fitting and a 22 gauge, 51 mm metal hub needle (Hamilton HAM191022) that is inserted into the two-stop tubing. This two-stop tubing is placed through the peristaltic pump, where it is then connected to an additional length of 2.79 mm PharMed BPT tubing and another 18-gauge stainless-steel needle. This needle is then placed directly into the bioreactor, with the tip dispensing medium directly into the culture at a continuous flow of 0.3 mL/h. All species were inoculated into the bioreactors at an initial cell concentration of 1 × 10^7^ cells/mL from an overnight preculture. The preculture was first centrifuged and resuspended in MBL with no carbon to minimize any nutritional carryover.

### Coculture Device.

The membrane separated coculture device was manufactured according to the previously published design, CAD files, and assembly instructions (49). This previous publication contains detailed instructions and video of how to assemble the device. The polycarbonate membranes (Isopore™ Membrane Filter, 0.1 μm VCTP; EDM Millipore) separated two cultures each containing 2.5 mL of chitin MBL. All cells were inoculated at an initial density of 1 × 10^7^ cells/mL and sealed with an adhesive gas-permeable seal (Thermo AB-0718). The entire device was placed on an orbital mini shaker at 300RPM (VWR 444-0269) in a 25 °C controlled room. OD_600_ was measured by withdrawing 100 μL from each culture, centrifuging at 1,000 rcf for 30 s, and measuring the OD_600_ of the supernatant in a 384-well plate.

### Measuring GlcNAc Consumption.

To quantify GlcNAc, we employed an Agilent 6545 LC-QTOF-MS in negative mode with high-sensitivity slicer, using a scan rate of 2 Hz, a mass range of 50 to 1,700 m/z, and a fragmentor voltage of 110 V. The drying gas flow was set to 10 L/min, nebulizer pressure to 35 psig, skimmer voltage to 65 V, and gas temperature to 320 °C. Separation was achieved using an Agilent HILIC-Z Poroshell column (100 mm × 2.1 mm, 1.8 µm particles). Mobile phase A consisted of LC–MS grade water with 10% acetonitrile and 0.3% ammonium hydroxide, while mobile phase B contained 90% acetonitrile. Samples were diluted 20-fold in 50% acetonitrile, and 4 µL was injected. The gradient started with 0% phase A, increased to 20% over 1 min, held for 30 s, and then returned to 0% for equilibration until 5 min. Quantification was performed using Agilent Quantitative software, based on the intensity of peaks compared to standards with known GlcNAc concentrations, using the chloride adduct (256.058 m/z) for detection.

### Exponential Growth Model.

To characterize bacterial growth kinetics to estimate time to reach OD_600_ 0.25, we fit an exponential growth model to experimentally determined OD_600_ data for cultures in log phase. Log phase is the period of growth in which the log transformed growth data is linear for at least four consecutive time points with a goodness of fit (R^2^) greater than 0.95. The growth model is described by the equation:

$$\mathrm{OD}(t)={\mathrm{OD}}_{0}\times e^{\mathrm{rt}},$$

where OD(t) is the OD600 at time t, OD_0_ is the OD600 at the beginning of log phase growth, and r is the growth rate.

### Microscope Images.

Samples (200 μL) were taken from exponentially growing *Vib*1A01 or *Psy*6C06 colloidal chitin cultures at similar planktonic ODs (~0.4). Chitin flakes were stained with 1 μg/mL FITC-WGA and cells were stained with a membrane dye FM 4-64 with a concentration of 5 μg/mL. The sample was then transferred to a glass bottom chamber for imaging with a Leica TCS SP8 inverted confocal microscope. The WGA signal was read in the GFP channel, which was excited with a 488 nm diode laser and the FM 4-64 was read in the mCherry channel and excited with a 580 nm diode laser. Fluorescence for both channels was detected through a 40×/1.3 objective and a highly sensitive HyD SP GaAsP detector.

### Chitinase Purification and Supplementation.

*Psy*6C06 was streaked from −80 °C glycerol stocks onto 1.5% agar plates containing MB 2216 (Fisher) medium. An overnight preculture was inoculated from a single colony in MB 2216. The following day, a 1% inoculum of this preculture was added to 400 mL of MBL medium with 2 g/L colloidal chitin and stirred at 200 rpm until the culture reached the early stationary phase. Following growth, the culture medium was centrifuged at 2,800 rcf for 20 min to remove residual chitin and cells, and then sterile filtered using a 0.2 µm membrane. The supernatant was concentrated 10-fold using an Amicon stirred cell (Millipore) with a 3 kDa cutoff filter. A protease inhibitor (Roche cOmplete EDTA-free protease inhibitor cocktail) was added to the final concentrate. The resulting solution was aliquoted into 500 µL portions in 1.5 mL microcentrifuge tubes, snap frozen in liquid nitrogen, and stored at −80 °C until use. The protein concentration in the chitinase enzyme cocktail was determined to be 0.07 mg/mL using the Bradford reagent. When used to supplement cultures during growth on chitin, enzyme was added to the inoculum at a 1% concentration from this stock.

## Supplementary Material

Appendix 01 (PDF)

Dataset S01 (CSV)

Movie S1. Confocal time-lapse of *Vib*1A01 interacting with colloidal chitin. Fluorescent staining highlights chitin particles (green, FITC-WGA) and bacterial membranes (red, FM 4–64).

Movie S2. Confocal time-lapse of *Psy*6C06 interacting with colloidal chitin. Fluorescent staining highlights chitin particles (green, FITC-WGA) and bacterial membranes (red, FM 4–64).

## Data Availability

Metabolomics data have been deposited in MassIVE (MSV000093541) (68). All other data are included in the manuscript and/or supporting information.
