# Supplementary material for: Hypersensitivity of chitin degradation to initial species densities due to monomer diffusion
Source: Proc Natl Acad Sci U S A. 2026 Jan 5;123(2):e2512676123. doi: 10.1073/pnas.2512676123 (PMC12799147; doi:10.1073/pnas.2512676123)
Supplement: Supplementary file 1 — Appendix 01 (PDF) [file pnas.2512676123.sapp.pdf]

Supplemental Information

Hyper-sensitivity of chitin-degradation to initial species densities due to monomer diffusion

Sammy Pontrelli[§], Ghita Guessous[§], Julian Trouillon, Aswin Krishna, Terence Hwa, and Uwe Sauer

[§]Authors contributed equally to this work

Species	Taxonomic ID	Functional Guild	NCBI BioProject	NCBI BioSample
AltA3R04	Alteromonas sp.	Exploiter	PRJNA478695	SAMN29350919
AmphC1R06	Amphritea sp.	Scavenger	PRJNA478695	SAMN29350932
ColC2M11	Colwellia psychrerythraea	Exploiter	PRJNA478695	SAMN29350933
MarD2M19	Marinobacter sp.	Scavenger	PRJNA478695	SAMN29350941
MarF3R08	Marinobacter sp.	Scavenger	PRJNA478695	SAMN29350954
MarF3R11	Marinobacter sp.	Scavenger	PRJNA478695	SAMN09522136
ParaC2R09	Paracoccus kamogawaensis	Scavenger	PRJNA478695	SAMN29350935
PhaB3M02	Phaeobacter sp.	Exploiter	PRJNA478695	SAMN09522130
Psy6C06	Psychromonas sp.	Degrader	PRJNA414740	SAMN08130274
SilA3R06	Silicibacter sp.	Exploiter	PRJNA478695	SAMN29350920
Vib1A01	Vibrio splendidus	Degrader	PRJNA414740	SAMN07809270
VibC3R12	Vibrio sp.	Exploiter	PRJNA478695	SAMN09522132
VibG2R10	Vibrio sp.	Degrader	PRJNA478695	SAMN29350956
VibI3M07	Vibrio sp.	Degrader	PRJNA478695	SAMN29350961

Table S1: Species used in this study and NCBI accession numbers for genomic sequences.

Species	CFU/ml/OD
**AltA3R04**	1.71E+09
**AmphC1R06**	2.72E+08
**ColC2M11**	7.01E+08
**MarD2M19**	4.04E+08
**MarF3R08**	1.14E+09
**MarF3R11**	1.30E+09
**ParaC2R09**	7.63E+08
**PhaB3M02**	7.23E+08
**Psy6C06**	5.06E+08
**SilA3R06**	1.89E+09
**Vib1A01**	4.15E+08
**VibC3R12**	3.56E+08
**VibG2R10**	3.52E+08
**VibI3M07**	8.32E+08

Table S2: CFU/mL/OD values for each species grown in MB2216 precultures. These values are used to determine the dilution required for precultures before inoculating experimental cultures that require consistent cell densities such as in Fig. 1A and 3E.

Degrader	Monoculture	Exploiter				
		AltA3R04	ColC2M11	PhaB3M02	SilA3R06	VibC3R12
Vib1A01	1.9	1.9	NA	1.9	1.9	3.51
VibG3R10	2.88	3.83	NA	4.9	2.88	4.68
VibI3M07	1.9	2.88	NA	2.88	3.83	4.9
Psy6C06	4.1	4.72	4.36	4.14	4.94	4

Degrader	Monoculture	Scavenger				
		AmphC1R06	MarD2M19	MarF3R08	MarF3R11	ParaC2R09
Vib1A01	1.33	1.33	2	2	2	1.1
VibG3R10	2.65	3.13	5.46	3.13	2.81	4.71
VibI3M07	2.65	2.19	3.13	2.5	3.13	3.13
Psy6C06	4.21	4	4.42	4.14	5.33	4.42

Table S3: Lag times (in days) of mono- and cocultures of degraders with exploiters (top) or scavengers (bottom). NA denotes cultures that showed no growth. Red boxes are cocultures with a delayed lag time compared to the monoculture greater than 12 hours. Green boxes are those with a decreased lag time compared to the monoculture greater than 12 hours.

Target	Direction	Sequence
1A01	F	GACAAGGACAGTGGCTCACA
1A01	R	GCTGCGAGTGATCAAGCATG
6C06	F	AGCACACCCTCGCTCTAAAC
6C06	R	TGAGAAAGTCCTGTTCCGCC
C2M11	F	GCAAGCGTTAACAAAAGCGC
C2M11	R	CCATGGTGTCGTTAGGCGTA
C3R12	F	CAAAAATCGCCCCCGCTTAC
C3R12	R	ATCGTCCTGGATCGCCATTG

Table S4: qPCR primers used in this study

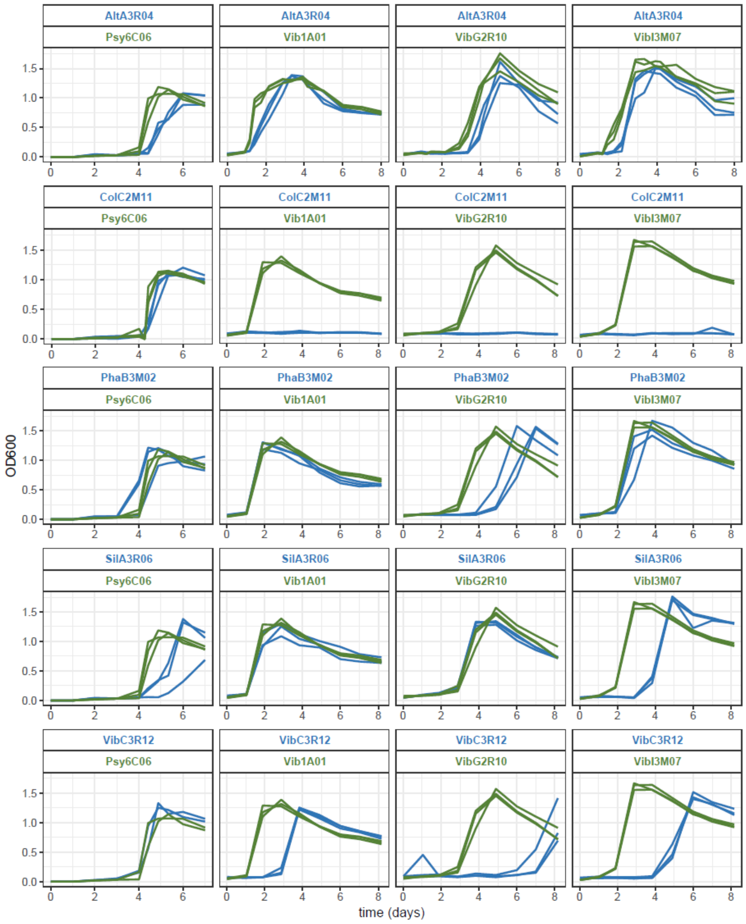

Figure S1: Growth of pairwise cocultures of each degrader (green text), and exploiter (blue text) on chitin as a sole carbon source. Green lines represent growth of degrader monocultures, blue is degrader:exploiter cocultures. All three biological replicates are shown. Each species is inoculated at a density of 10^7 cells/mL from a preculture of MB2216. The exact volumes needed are calculated using OD_{600} to cell density values from Table S2.

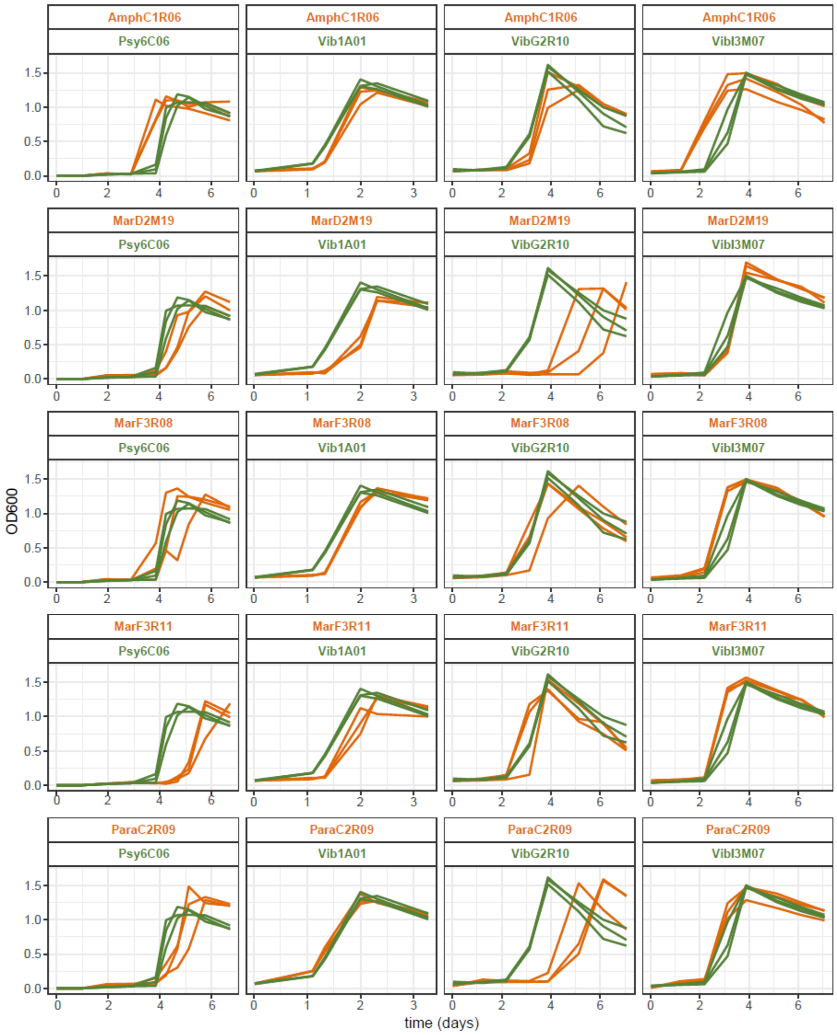

Figure S2: Growth of pairwise cocultures of degraders (green text), and scavengers (orange text) on chitin as a sole carbon source. Green lines represent growth of degrader monocultures, orange is degrader:scavenger cocultures. All three biological replicates are shown. Each species is inoculated at a density of 10^7 cells/mL from a preculture of MB2216. The exact volumes needed are calculated using OD_{600} to cell density values from Table S2.

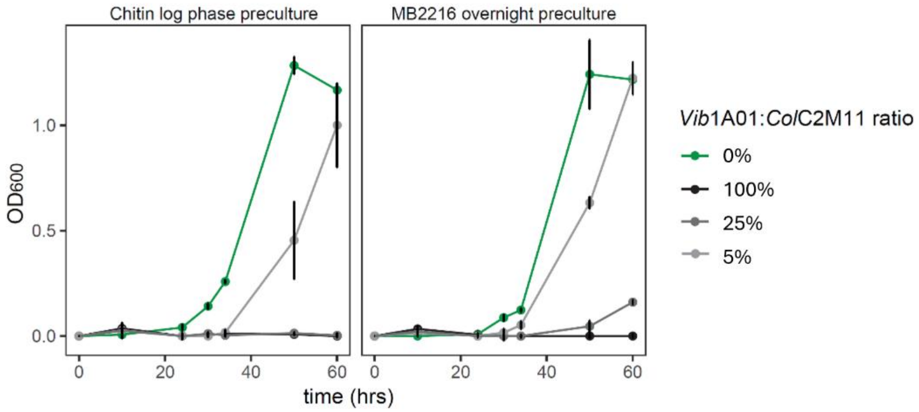

Figure S3: Growth on colloidal chitin of *Vib*1A01 monoculture or coculture with *Col*C2M11 with different inoculation ratios or preculture conditions. Precultures were taken from *Vib*1A01 growing in MB2216 rich medium overnight culture or from log phase growth on colloidal chitin. In all cases, *Col*C2M11 is taken from a MB2216 overnight preculture. Experiments were performed in triplicate, and error bars represent standard deviation from the mean. In all cases, *Vib*1A01 is inoculated at $1x10^7$ cells/mL.

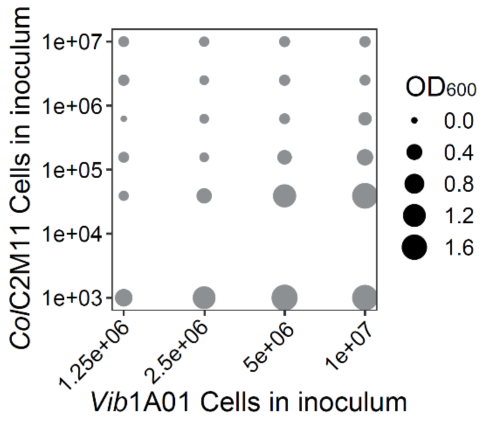

Figure S4. Growth of *Vib*1A01 cocultures with *Col*C2M11 on colloidal chitin at different initial cell ratios at 44 hours. The experiment was performed in triplicate.

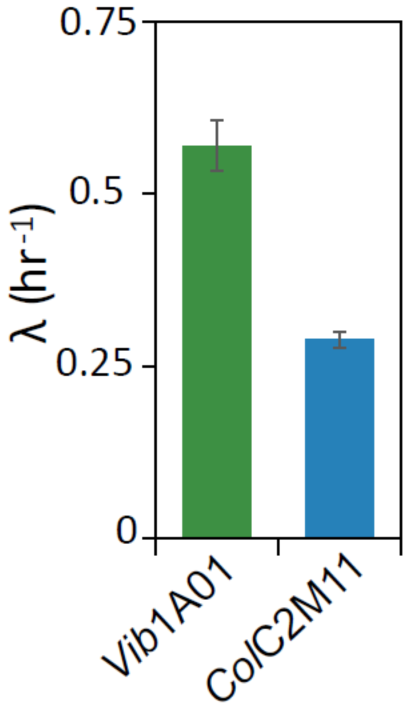

Figure S5: Maximum growth rates of *Vib*1A01 and *Col*C2M11 on 20mM GlcNAc in a batch culture. Error bars are standard deviation of three replicate cultures.

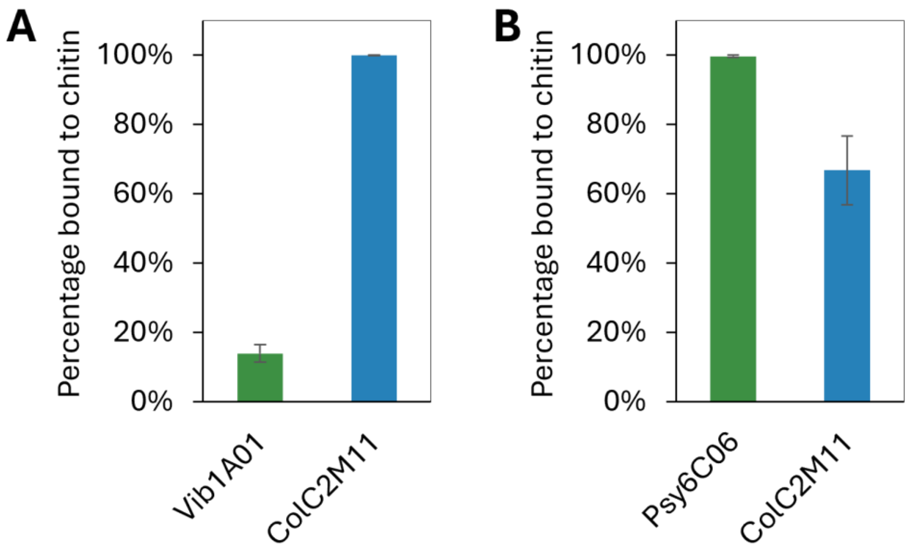

Figure S6: Distribution of degrader and exploiter cells between planktonic and particle-bound fractions in cocultures. A) *Vib*1A01 and *Col*C2M11 at 24 hours and B) *Psy*6C06 and *Col*C2M11 at 72 hours. These timepoints correspond to the last sample taken before a visible increase in OD_{600} in the planktonic phase. Species abundances in each fraction were quantified by qPCR from biological triplicates, with each sample measured in technical duplicate. Error bars show the standard deviation across biological replicates.

Supplemental Videos

Video S1: Confocal time-lapse of *Vib*1A01 interacting with colloidal chitin. Fluorescent staining highlights chitin particles (green, FITC-WGA) and bacterial membranes (red, FM 4-64).

Video S2: Confocal time-lapse of *Psy*6C06 interacting with colloidal chitin. Fluorescent staining highlights chitin particles (green, FITC-WGA) and bacterial membranes (red, FM 4-64).

Supplementary Note 1: Direct inhibition via the release of toxic compounds by the exploiters

In order to decouple the effect of metabolic competition from other mechanisms of direct inhibition such as toxic compound secretion by the exploiters, we directly tested whether the exploiters secreted any toxic compounds that affected the growth of the degraders. To do this, we set on to test whether the coculture extracted supernatant had an inhibitory effect on degrader monocultures **(Fig. S7)**. However, to remove any confounding factors that may arise from compounds secreted by the degraders themselves, we also extracted the monoculture's supernatant as a point of comparison. If the exploiter secretes a toxin, we expect the degrader to exhibit a diminished growth phenotype in the coculture compared to the monoculture extracted supernatant.

The two degraders *Psy*6C06 and *Vib*1A01 were grown on colloidal chitin in mono- and coculture with their paired exploiters until early stationary phase. Note that *Col*C2M11 had to be inoculated at 20-fold lower density than *Vib*1A01 for the coculture to grow; see **Fig. 1D**. Cell- and chitin-free supernatants were obtained by filtration and diluted 50% with fresh colloidal chitin medium. A toxin-producing exploiter would therefore lead to suppressed degrader growth in coculture compared to monoculture controls.

We found that while the exploiters *VibC2M11* and VibC3R12 inhibited the degrader *Vib1A01* in fresh media (second column, **Fig. 1C)**, the addition of the monoculture and coculture extracted supernatants to *Vib1A01* monocultures resulted in similar Vib1A01 monoculture growth curves (**Fig. S8 middle and left column**), indicating that no toxin was present. The inhibition observed for *Vib1A01* by *VibC2M11* and VibC3R12 is thus likely not a result of toxin secretion.

On the other hand, the addition of coculture extracted supernatant from a *Psy6C06:AltA3R04* coculture significantly inhibited the growth of *Psy6C06* monocultures. The degrader monoculture reached less than half the maximum density compared to when it was grown in fresh chitin medium, **(Fig. S8 right column).** This indicates that *Alt*A3R04 secretes an inhibitory compound. Filtration of the supernatant through a 10 kDa molecular weight cutoff filter does not prevent the inhibition, suggesting that *Alt*A3R04 releases a low molecular weight inhibitor **(Fig. S8, bottom row).**

Untargeted liquid chromatography quadrupole time of flight mass spectrometry (LC-QTOF-MS) revealed accumulation of 41 putatively annotated metabolites in coculture of *Psy*6C06 and *Alt*A3R04 compared to *Psy*6C06 monoculture on colloidal chitin **(Supplemental Dataset 1)**. Of interest is 6-aminopenicillanate (6-APA, **Fig. S9A**), a precursor or degradation product of β-lactam antibiotics[1], which also possesses antibiotic properties by disrupting cell wall biosynthesis[2,3] leading to cell lysis[4] in Gram-negative bacteria. Another metabolite of note is phosphorylated chitobiose; it is accumulated after 4 days (**Fig. S9B**), corresponding to the onset of growth of the culture (**Fig. S9B)**. Since *Alt*A3R04 is unable to take up chitobiose[5], the GlcNAc dimer, accumulation of phosphorylated chitobiose implies toxin-dependent cell lysis and release of intracellular chitin breakdown products from *Psy*6C06. We thus hypothesize that *Alt*A3R04 produces a β-lactam family antibiotic that inhibits *Psy*6C06 and possibly other degraders in cocultures **(Fig. 1C)**.

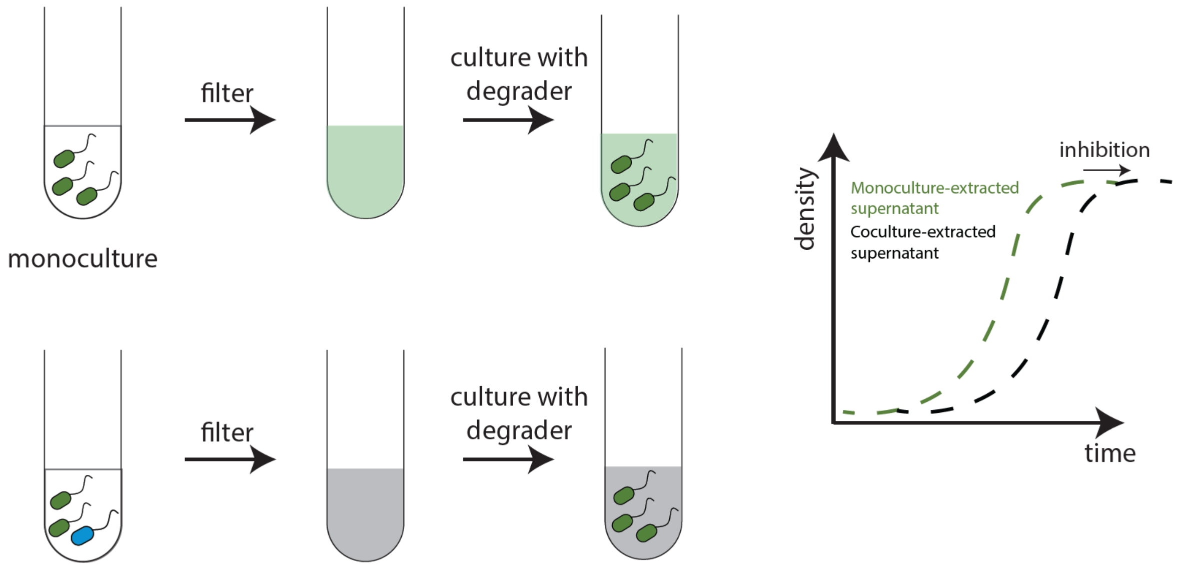

Fig S7: Experimental setup for detecting toxic compounds secreted by exploiters. To test the potential effect of toxin secretion by the exploiters on the degraders, cell and chitin-free supernatants were extracted from saturated chitin monocultures (light green) and cocultures (gray). Supernatants were mixed with diluted 50% with fresh colloidal chitin media and used as growth media to assess the growth of chitin degraders in monoculture. An inhibitory effect on the growth of the degraders indicates the presence of a toxin secreted by the exploiter.

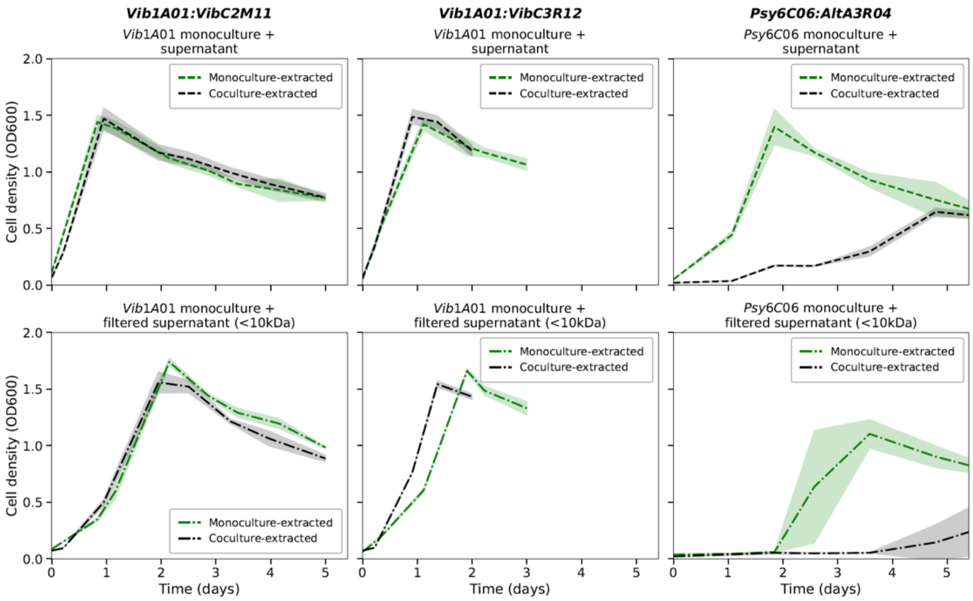

Figure S8: Growth curves of degraders in cell-free culture supernatants. All growth curves correspond to monoculture growth of degraders Vib*1A01* (left and middle) and Psy*6C06* (right) on colloidal chitin. Dashed lines in the first row correspond to the addition of 50% diluted supernatant derived from their paired exploiter (left to right: *VibC2M11, VibC3R12*, *Alt*A3R04) and dashed-dotted lines in the second row correspond to the addition of the same supernatant, passed through a 10kDa filter. Two types of supernatants were tested: one that was extracted from degrader monoculture (green), and another from degrader:exploiter cocultures (black), both grown on colloidal chitin. Supernatants were harvested at early stationary phase and degraders were inoculated immediately without freezing to prevent denaturation of potential toxins. Shaded areas represent the standard deviation from the mean of three biological replicates.

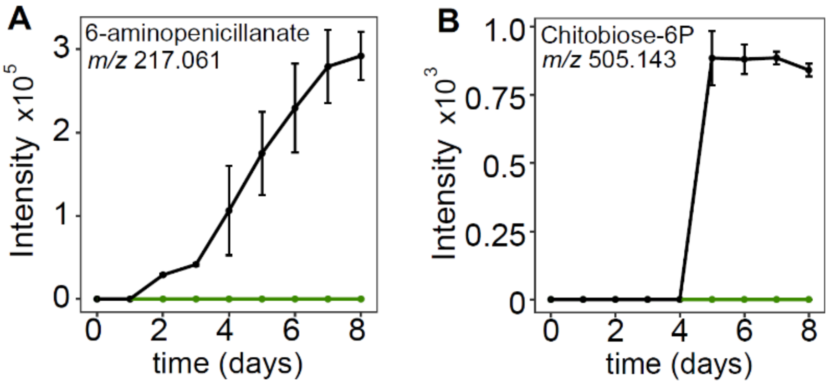

Figure S9: Accumulation of metabolites in *Psy*6C06 monoculture or *Psy*6C06:*Alt*A3R04 coculture. A) Metabolomics measurement of 6-aminopenecillanate in *Psy*6C06:*Alt*A3R04 coculture (black) and *Psy*6C06 monoculture (green) on colloidal chitin. B) Measurement of chitobiose-6-phosphate in *Psy*6C06:*Alt*A3R04 coculture (black) and *Psy*6C06 monoculture (green). Error bars represent the standard deviation from the mean of three biological replicates.

Supplementary Note 2: Nutrient competition in fed-batch culture

In this Note, we analyze the competition of two species growing on the same nutrient, GlcNAc, in a fed-batch culture. The result will be used to interpret the data of the fed-batch coculture of *Vib*1A01 and *Col*C2M11 as described in the main text.

1. Solution for the monoculture

We first describe the monoculture in the fed-batch reactor. Let $\rho(t)$ be the biomass density (or OD) of a single species of bacteria grown in the fed-batch culture, inoculated at ρ_0 at time $t = 0$. Let the nutrient concentration in the culture be $n(t)$, and the nutrient drip rate be α. Then the dynamics of the system are described by the following equations:

$$\frac{d}{dt}n = \alpha - r(n)\rho/Y \qquad \text{(N2.1.1)}$$

$$\frac{d}{dt}\rho = +r(n)\rho \qquad \text{(N2.1.2)}$$

where Y is the biomass yield of the nutrient, and

$$r(n) = r_{max}\frac{n}{n+K} \qquad \text{(N2.1.3)}$$

the Monod growth kinetics, with saturated growth rate r_{max}, and Monod constant K.

Eq. (N2.1.1) and (N2.1.2) give the condition,

$$\frac{d}{dt}\rho + Y\frac{d}{dt}n = \alpha Y \qquad \text{(N2.1.4)}$$

reflecting the constraint that the added nutrient amount is either converted to biomass or remains in the culture. If most of the nutrient is captured by the cells (as will be shown shortly below), then $|Y\frac{d}{dt}n|$ is negligible compared to $\frac{d}{dt}\rho$, and Eq. (N2.1.4) is easily solved, giving

$$\rho(t) \approx \rho_0 \cdot (1 + t/t_0) \qquad \text{(N2.1.5)}$$

where $t_0 \equiv \rho_0/(\alpha Y)$. Inserting the approximate solution (N2.1.5) into Eq. (N2.1.2) gives the dynamics of $n(t)$:

$$n \approx \frac{K}{r_{max} \cdot (t + t_0) - 1} \qquad \text{(N2.1.6)}$$

Since $\frac{d}{dt}\rho = \rho_0/t_0$ and $Y\frac{d}{dt}n = -n^2 Y r_{max}/K$, then the approximation $|Y\frac{d}{dt}n| \ll \frac{d}{dt}\rho$ requires that

$$t + t_0 \gg r_{max}^{-1} + \sqrt{K/(r_{max}\alpha)} \qquad \text{(N2.1.7)}$$

For $r_{max} \approx 0.6/h$, $K \approx 1\ \mu M$ (**Fig. S5**, and **Table S5** in **Supp Note 3**), and with the drip rate being $\alpha \sim 10\ \mu M/h$, the right hand side of the inequality is $\sim 1\ h$. For an initial inoculant at $\rho_0 = 0.01$ OD and an (inverse) yield being $Y^{-1} = 6$ mM GlcNAc/OD, we have $t_0 \sim 6\ h$. Thus, the condition (N2.1.7) is satisfied for all t, and the solutions (N2.1.5) and (N2.1.6) are almost exact.

From Eq. (N2.1.6), we note also that $\frac{n}{K} < \frac{1}{r_{max}t_0 - 1} < 1$. Hence, the Monod growth kinetics (N2.1.3) can be approximated by its linear form, i.e., $r(n) \approx r_{max}n/K$. This is the form we will use below. To simplify the notation, we define $\nu \equiv r_{max}/K$ such that $r(n) \approx \nu \cdot n$.

2. Two-species competition.

Now we consider two species (A and C), with biomass $M_A(t)$ and $M_C(t)$, respectively, whose growth rates are (in the linear approximation) $r_A(n) \approx \nu_A \cdot n$, $r_C(n) \approx \nu_C \cdot n$, with $\nu_A \equiv r_{max,A}/K_A$ and $\nu_C \equiv r_{max,C}/K_C$. The equations describing the two species dynamics are

$$\frac{d}{dt}\rho_A = \nu_A n \rho_A \tag{N2.2.1}$$

$$\frac{d}{dt}\rho_C = \nu_C n \rho_C \tag{N2.2.2}$$

$$\frac{d}{dt}n = \alpha - \nu_A n \rho_A/Y - \nu_C n \rho_C/Y \tag{N2.2.3}$$

where the same yield Y is taken for the two species for simplicity.

The total biomass density $\rho(t) \equiv \rho_A(t) + \rho_C(t)$ again satisfies the condition (N2.1.4), and with $\frac{d}{dt}n$ negligible, its solution is again the linear form given in Eq. (N2.1.5). To see how biomass is partitioned between the two species, we define the fractional biomass, $\phi_i(t) \equiv \rho_i(t)/\rho(t)$. In terms of ϕ_i, the dynamics of ρ can be written (from Eqs. (N2.2.1) and (N2.2.2)) as

$$\frac{d}{dt}\rho = \frac{d}{dt}\rho_A + \frac{d}{dt}\rho_C = (\nu_A\phi_A + \nu_C\phi_C)\, n \cdot \rho \tag{N2.2.4}$$

and the dynamics of ϕ_i are given as

$$\frac{d\phi_i}{dt} = \frac{1}{\rho}\frac{d\rho_i}{dt} - \frac{\rho_i}{\rho^2}\frac{d\rho}{dt} = \nu_i n \phi_i - (\nu_A\phi_A + \nu_C\phi_C)\, n\phi_i \tag{N2.2.5}$$

where we used Eqs. (N2.2.1), (N2.2.2) and (N2.2.4) in the last step above. Using $\phi_A + \phi_C = 1$, Eq. (N2.2.5) can be written as

$$\frac{d}{dt}\phi_A = (\nu_A - \nu_C) n \phi_A \phi_C \tag{N2.2.6}$$

$$\frac{d}{dt}\phi_C = (\nu_C - \nu_A) n \phi_A \phi_C \tag{N2.2.7}$$

or in terms of the fractional difference, $\Delta\phi \equiv \phi_C - \phi_A$,

$$\frac{d}{dt}\Delta\phi \ = \tfrac{1}{2}\Delta\nu \cdot n(t) \cdot (1 - (\Delta\phi)^2) \tag{N2.2.8}$$

where $\Delta\nu \equiv \nu_C - \nu_A$.

To solve Eq. (N2.2.8) and obtain the dynamics of $\Delta\phi$, we need to know the form of $n(t)$. This can be obtained by exploiting Eq. (N2.2.4). Writing $\nu_A\phi_A(t) + \nu_C\phi_C(t) = \bar{\nu} + \tfrac{1}{2}\Delta\nu\Delta\phi(t)$ where $\bar{\nu} \equiv \tfrac{1}{2}(\nu_A + \nu_C)$, we have

$$n(t) = \frac{\frac{d}{dt}\rho/\rho}{\bar{\nu} + \frac{1}{2}\Delta\nu\Delta\phi(t)} = \frac{1}{\bar{\nu} + \frac{1}{2}\Delta\nu\Delta\phi(t)} \cdot \frac{1}{t + t_0} \tag{N2.2.9}$$

where we used the solution (N2.1.5) for $\rho(t)$. Inserting this form of $n(t)$ in Eq. (N2.2.8), we obtain a closed ODE for $\Delta\phi(t)$,

$$(\beta + \Delta\phi(t))\frac{d}{dt}\Delta\phi \ = (1 - (\Delta\phi)^2)/(t + t_0) \tag{N2.2.10}$$

with $\beta \equiv 2\bar{\nu}/\Delta\nu = \frac{\nu_C + \nu_A}{\nu_C - \nu_A}$.

Eq. (N2.2.10) can be integrated exactly:

$$\int d(\Delta\phi)\frac{\beta + \Delta\phi}{1 - (\Delta\phi)^2} \ = \int \frac{dt}{t + t_0} \tag{N2.2.11}$$

leading to the solution (for initial condition $\Delta\phi(0) = 0$, i.e., $\phi_A(0) = \phi_C(0) = 0.5$):

$$\frac{(1 + \Delta\phi)^{\frac{\beta - 1}{2}}}{(1 - \Delta\phi)^{\frac{\beta + 1}{2}}} = 1 + \frac{t}{t_0} \tag{N2.2.12}$$

or expressed more transparently as

$$\frac{1 + \Delta\phi}{(1 - \Delta\phi)^{\eta}} = \left(1 + \frac{t}{t_0}\right)^{\eta - 1} \tag{N2.2.13}$$

where $\eta \equiv \nu_C/\nu_A$, a dimensionless ratio of the Monod parameters, gives the ratio of growth rates (i.e., $\eta = r_C/r_A$) at low nutrient concentrations where $r_i = \nu_i n$.

To see the form of the solution described by Eq. (N2.2.13), let us suppose $\eta > 1$. Then in the long time limit ($t \gg t_0$) where the right-hand side diverges, the left-hand side must also diverge, which happens

for $\Delta\phi = \phi_C - \phi_A \to 1$ (corresponding to $\phi_C \to 1$ and $\phi_A \to 0$). The asymptotic solution can be written as $\Delta\phi(t) \approx 1 - 2^{1/\eta}(t/t_0)^{-(\eta-1)/\eta}$, or $\phi_A(t) = \frac{1}{2}(1-\Delta\phi(t)) \approx (2t/t_0)^{(\nu_A-\nu_C)/\nu_C}$, i.e., the slower-growing species, in this case, species A because we took $\eta > 1$ ($\nu_C > \nu_A$), is depleted from the coculture in a power-law fashion, with an exponent that is proportional to the difference in the growth parameters.

Alternatively, the limit that η is slight larger than 1, i.e., for $\eta - 1 \ll 1$, such that $\Delta\phi \ll 1$ also, Eq. (N2.2.13) can be simplified to

$$\Delta\phi \approx \frac{\eta-1}{2} \cdot \ln\left(1+\frac{t}{t_0}\right) \qquad \text{(N2.2.14)}$$

3. Quantitative comparison to experimental results.

The calculation in the previous sections assumed that the volume of the culture is unchanged. However, to compare to the experimental data quantitatively, we need to incorporate the fact that in the fed-batch reactor, the addition of GlcNAc does change the culture volume. According to **Method**, the flow to the bioreactor is $\alpha_V = 0.3\ ml/h$. For a culture with initial volume of $V_0 = 20\ ml$, the volume changes as $V(t) = V_0 + \alpha_V t$, with an increase of ~20ml over the course of the 72 h experiment. The volume change affects the GlcNAc concentration $n(t)$ and cell densities $\rho_i(t)$. To treat this effect, we introduce the GLcNAc amount $m(t)$, with $n(t) = m(t)/V(t)$, and the biomass of each species, $M_i(t)$, with $\rho_i(t) = M_i(t)/V(t)$. Eqs. (N2.2.1)-(N2.2.3) are then modified to

$$\tfrac{d}{dt}M_A = \nu_A \cdot (m/V)M_A \qquad \text{(N2.3.1)}$$

$$\tfrac{d}{dt}M_C = \nu_C \cdot (m/V)M_C \qquad \text{(N2.3.2)}$$

$$\tfrac{d}{dt}m = \alpha_m - \nu_A \cdot (m/V)M_A/Y - \nu_C \cdot (m/V)M_C/Y \qquad \text{(N2.3.3)}$$

Since GlcNAc inflow is at 1mM, the rate of GlcNAc increase is $\alpha_m = 1mM \cdot 0.3mL\,h^{-1} = 0.3\ \mu mol\ h^{-1}$. The mass conversation condition becomes $\frac{d}{dt}M_A + \frac{d}{dt}M_C + Y^{-1}\frac{d}{dt}m = \alpha_m$. Neglecting $\frac{d}{dt}m$ as before, we obtain a simple relation for the total biomass $M(t) \equiv M_A(t) + M_C(t)$:

$$M(t) \approx M_0 \cdot (1 + t/t_0) \qquad \text{(N2.3.4)}$$

where $t_0 \equiv M_0/(\alpha_m Y)$. For the coculture with initial inoculant of each species at OD = 0.01 and an initial reaction volume $V_0 = 20\ ml$, we have $M_0 = 0.4$ OD·ml. This gives $t_0 = 8\ h$.

The dynamics of $\phi_i = M_i/M$ can be derived as in Sec. 2, leading to Eqs. (N2.2.6)-(N2.2.8), with $n(t)$ replaced by $m(t)/V(t)$, and with

$$\tfrac{d}{dt}M = \tfrac{d}{dt}M_A + \tfrac{d}{dt}M_C = (\nu_A\phi_A + \nu_C\phi_C) \cdot (m/V)\ M \qquad \text{(N2.3.5)}$$

Then, Eq. (N2.3.5) can be used to solve for the factor m/V, with

$$\frac{m(t)}{V(t)} = \frac{\frac{d}{dt}M/M}{\bar{\nu} + \frac{1}{2}\Delta\nu\Delta\phi(t)} = \frac{1}{\bar{\nu} + \frac{1}{2}\Delta\nu\Delta\phi(t)} \cdot \frac{1}{t + t_0} \qquad \text{(N2.3.6)}$$

Thus, the final equation for $\Delta\phi$ is the same as Eq. (N2.2.10), with the same solution (N2.2.13) [and its approximation (N2.2.14)], except that the value of t_0 is given by $\rho_0 V_0/(\alpha_m Y)$; in other words, the GlcNAc influx rate α of the constant volume scenario considered in Sec. 1 and 2 is replaced by α_m/V_0.

In **Fig. S10A**, we plot the dependence of $\Delta\phi$ vs η according to solution Eq. (N2.2.13) for several values of the fed-batch runtime t and for $t_0 = 8\ h$ (solid lines). For $t = 72\ h$ (black line), we compare the analytical solution to the exact numerical solution calculated from Eqs. (N2.3.1)-(N2.3.3) (black circles). The analytical solution is seen to be in quite good agreement with the exact numerical solution. **Fig. S10B** shows a zoomed in view for nutrient uptake ratio $\eta \approx 1$. The dashed line is the approximate solution given in Eq. (N2.2.14).

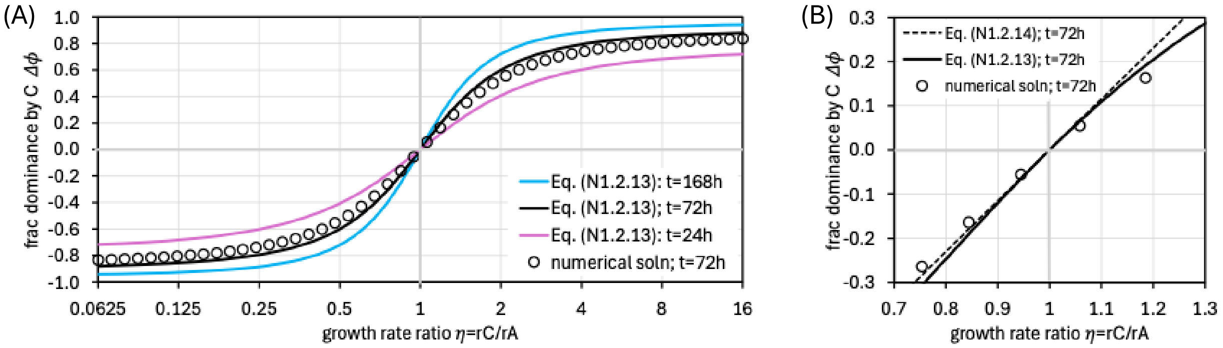

Figure S10: Comparing the analytical and numerical solutions of the system as defined by Eq. (N2.3.1)-(N2.3.3). Plotted is the dependence of the fractional species dominance $\Delta\phi \equiv (M_C - M_A)/(M_C + M_A)$ for various values of $\eta \equiv \left(r_{max,C}/K_C\right)/\left(r_{max,A}/K_A\right)$, which gives the growth rate ratio $r_C : r_A$ at low nutrient concentrations. **(A)** Solutions for various duration of fed-batch growth t, with $t_0 = 8h$. Solid lines of different colors show the analytical solution Eq. (N2.2.13), which can be rewritten as $\eta = \ln[(1 + t/t_0)(1 + \Delta\phi)]/\ln[(1 + t/t_0)(1 - \Delta\phi)]$. Note that the solution exhibits the built-in symmetry that for $\Delta\phi \to -\Delta\phi$, $\eta \to 1/\eta$, which arises from switching the species labels A and C. Open circles are the result of direct numerical solution. **(B)** Solution for systems with comparable uptake ($\eta \approx 1$) after 72h of fed-batch growth, with the approximate solution Eq. (N3.1.14) shown as the dashed line.

Finally, we use the solution to extract the value of η from our fed-batch data: The data in **Fig. 2B** of the main text shows that the abundance of Vib1A01 is about 2/3 of that of ColC2M11 at 72h, i.e., $\phi_A \approx \frac{2}{3}\phi_C$; this leads to $\phi_A \approx 40\%$, $\phi_C \approx 60\%$, or $\Delta\phi \approx 20\%$. For $t = 72\ h$, $t_0 = 8\ h$, Eq. (N2.2.14) becomes $\Delta\phi \approx 1.15 \cdot (\eta - 1)$. Then for $\Delta\phi \approx 20\%$, we have $\eta \approx 1.2$; see **Fig. S10B**.

Supplementary Note 3: Effect of nutrient competition on chitin degradation

In this Note, we describe a mathematical model describing the competitive metabolic interactions in a coculture between a chitin degrader (*Vib*1A01, species A), which produces and excretes chitinases, resulting in the release of labile nutrients (GlcNAc), and an exploiter (*Col*C2M11, species C), which competes with the degrader in the uptake of the labile nutrient. Our goal is to demonstrate that a metabolism-based model can recapitulate the drastic effect observed in the main text: that the addition of a small amount (10%) of the exploiters can inhibit growth (**Fig. 1D** of the main text) even though the exploiters do not exhibit much stronger affinity for GlcNAc (**Supp. Note 2** and **Fig. 2**). We will show that the sensitivity to exploiters is possible in the context of an incipient Allee transition for the monoculture. In this note, we follow the model and notation introduced in Guessous et al.[6] describing the coupled dynamics of the degraders, chitinases, and labile nutrients, but include the additional effect of nutrient uptake by the exploiters.

1. Nutrient dynamics

We start by writing down the flux balance equation describing the generation, uptake, and diffusion of the labile nutrient (GlcNAc), whose concentration is denoted by $n(R,t)$ around the surface of a spherical chitin particle of radius R_0:

$$\frac{\partial}{\partial t}n = D_n\nabla^2 n + \left[\kappa_E\epsilon(t) - J_{uptake}(n)\right]\cdot\delta(R-R_0) \qquad \text{(N3.1.1)}$$

Here, R is the distance from the center of the chitin particle, D_n is the nutrient diffusion coefficient, $\epsilon(t)$ is the chitinase amount per surface area, and κ_E is the rate of nutrient generation per chitinase (so that $\kappa_E\epsilon$ is the nutrient generated by chitinases per surface area). The 2nd term in the square bracket is the total flux of nutrient uptake by cells on the particle surface. Describing the replication rate of each species i by the Monod growth kinetics, $r_i(n) = r_{max,i}\cdot n/(n+K_i)$ where $r_{max,i}$ is the batch culture growth rate and K_i is the Monod constant, then

$$J_{uptake}(n) = r_A(n)\sigma_A(t)/Y + r_C(n)\sigma_C(t)/Y \qquad \text{(N3.1.2)}$$

where $\sigma_i(t)$ is the number of particle-attached cells of species i per surface area and Y is the biomass yield (taken to be the same for the two species).

Several key approximations are taken in the formulation of the dynamics:

- Nutrient uptake occurs only on the particle surface; this is justified by the drop in nutrient concentration away from the particle and is well-established numerically and experimentally in Guessous et al.[6]
- Cells and chitinases are assumed to be distributed uniformly across the particles (so that σ_i and ϵ are independent of the location on the particle). This assumption, taken for mathematical convenience, may be justifiable when both cell and chitinase densities increase. However, it is an oversimplification during the early stages when there are only a few cells on particles.

Eqs. (N3.1.1) and (N3.1.2) are supplemented by the no-flux boundary condition at the particle surface,

$$-D_n \frac{\partial n}{\partial R}\bigg|_{R=R_0} = \kappa_E \epsilon(t) - J_{uptake}\big(n_s(t)\big) \quad \text{(N3.1.3)}$$

where $n_s(t) \equiv n(R_0, t)$ is the nutrient concentration at the particle surface, whose value determines the replication rates of the degraders and exploiters on the surface of the chitin particles, $r_A(n_s)$ and $r_C(n_s)$ respectively.

Since the dynamics of nutrient production and uptake happen on timescales, much faster than those of enzyme production and cell growth, we take the approximation that $\frac{dn_s}{dt} = 0$ and look for the quasi-steady-state solution n_s for given values of σ_A, σ_C, and ϵ: Since the replication rates r_A, r_C are much below the batch culture growth rates $r_{max,A}$, $r_{max,C}$, respectively, the Monod kinetics can be approximated by the linear form $r_i(n) = r_{max,i} \cdot n/K_i$. In this case, the steady-state solution to Eq. (N3.1.1) becomes the 1/R-Coulomb potential, $n(R,t) = n_s(t) \cdot R_0/R$, and the boundary condition Eq. (N3.1.3) leads to an important relation between n_s and the values of σ_A, σ_C, ϵ:

$$n_s(t) \cdot \left(\frac{D_n}{R_0} + \frac{r_{max,A}\sigma_A(t)}{K_A Y} + \frac{r_{max,C}\sigma_C(t)}{K_C Y}\right) = \kappa_E \epsilon(t) \quad \text{(N3.1.4)}$$

The time dependence indicated in these quantities refers to their inter-relationship as their quantities change over the scale of the cell doubling time. Note the distinction from the $\frac{\partial n_s}{\partial t} = 0$ limit taken for Eq. (N3.1.1), which refers to the time scale of nutrient diffusion.

In Eq. (N3.1.4), the first time can be identified as the flux of nutrient loss, or leakage, due to diffusion, whose unit is the number of GlcNAc molecules per surface area per time. Note that this term is proportional to D_n as expected of diffusive loss, and inversely proportional to R_0, reflecting a dependence on local geometry: the larger the curvature, the larger the leakage. To make contact with experimentally relevant quantities, it is helpful to convert the above formulation on a single chitin particle to bulk quantities involving the number of chitin particles (assumed to be of identical size R_0) per volume, ρ_{chitin}. Let the chitinase "concentration" be $E = \epsilon \cdot 4\pi R_0^2 \rho_{chitin}$, and the surface-attached cell density be $A \equiv \sigma_A \cdot 4\pi R_0^2 \rho_{chitin}$, $C \equiv \sigma_C \cdot 4\pi R_0^2 \rho_{chitin}$. Multiplying $4\pi R_0^2 \rho_{chitin}$ to the two sides of Eq. (N3.1.4), we obtain

$$n_s(t) \cdot \left(\gamma + \frac{r_{max,A}A(t)}{K_A Y} + \frac{r_{max,C}C(t)}{K_C Y}\right) = \kappa_E E(t) \quad \text{(N3.1.5)}$$

with a leakage rate given by $\gamma = \frac{D_n}{R_0} \cdot 4\pi R_0^2\, \rho_{chitin}$. Let $\phi_{chitin} \equiv \frac{4\pi}{3} R_0^3\, \rho_{chitin}$ be the chitin volume fraction, given by the total chitin amount through the chitin density $d_{chitin} \approx 1.4\ g/cm^3$. For a total

amount of 0.2%w/v, we find that $\phi_{chitin} \cdot d_{chitin} = 0.002 g/cm^3$, or that the volume fraction $\phi_{chitin} \approx 0.14\%$. For fixed ϕ_{chitin}, we see that the leakage rate

$$\gamma = \frac{3D_n}{R_0^2}\phi_{chitin} \tag{N3.1.6}$$

is inversely proportional to R_0^2.

Comparing the situation studied by Guessous et al.[6] (chitin chips where $R_0 \approx 150\ \mu m$) to the current work (colloidal chitin particles where $R_0 \approx 10\ \mu m$), we find that the ratio of radii being 15x results in a leakage rate, γ, which is 225x larger for colloidal chitin for the same chitin density ϕ_{chitin}. Numerically, for 0.2%w/v chitin used in the current work, $\gamma = \frac{3\times 500 \mu m^2/s}{(10\ \mu m)^2} \times 0.14\% = 0.021/s = 76/h$ whereas for chitin chips in Guessous et al.[6], γ is only 0.3/h.

Eqs. (N3.1.5) and (N3.1.6) are the main take away of this analysis of nutrient flux balance at the particle surface. Eq. (N3.1.5) is a condition relating the nutrient concentration on the surface to the density of cells and enzymes, and Eq. (N3.1.6) gives the magnitude of the nutrient leakage rate γ. The nutrient concentration $n_s(t)$ is fixed by macroscopic balances through the population growth rate as discussed below.

For a growing culture, $A(t)$ and $E(t)$ increase exponentially in steady state growth, and the nutrient leakage term γ is negligible. However, nutrient leakage can be important at low chitinase and cell densities during the initial phase of growth on chitin, especially for small chitin particles where the leakage rate γ is large. It is useful to re-express Eq. (N3.1.5) in a way that makes the leakage rate γ more directly meaningful. Factoring out a factor $r_{max,A}/(K_A Y)$ from Eq. (N3.1.5), we have

$$r_A(n_s) \cdot \left(\rho_{leak} + A(t) + \eta C(t)\right) = \kappa_E E(t) Y \tag{N3.1.7}$$

where

$$\rho_{leak} \equiv \gamma K_A Y / r_{max,A} \tag{N3.1.8}$$

$$\eta \equiv \left(r_{max,C}/K_C\right) / \left(r_{max,A}/K_A\right) \tag{N3.1.9}$$

The term ρ_{leak} in Eq. (N3.1.7) expresses the leakage term as an effective cell density. It can be thought of as an additional population of degraders that the enzymes need to support through their generation of labile nutrient (even in the absence of the exploiters). It gives the scale of the exploiter density above which leakage can be neglected.

Numerically, taking the Monod constant[◊] to be $K_A \approx 1\ \mu M$, $r_{max,A} \approx 0.6/h$, and the GlcNAc yield as 1 OD/ml/6mM (**Fig. S5**, **Table S5**), we obtain $\rho_{leak} \approx 0.02\ OD$, or 2×10^7 cells/ml, for the colloidal

[◊] The Monod constant K_A was indirectly estimated in Guessous et al[6], and further confirmed here by direct measurement of the residual GlcNAc concentration for a Vib1A01 monoculture grown in GlcNAc; see **Fig. S11**.

chitin particles. Thus, the leakage flux is a substantial factor that needs to be considered for the range of cell densities that we study (where the initial inoculant used was 0.01 OD). Finally. Eq. (N3.1.7) also shows that the presence of the exploiters, at density C, serves effectively as an additional leakage term that increases ρ_{leak} by an amount ηC. The factor η, which describes the ratio of growth rates (i.e., $\eta \approx r_C : r_A$) at low nutrient concentrations where the Monod growth is linear, is close to 1 due to the similarity in growth characteristics; see **Supplementary Note 2**.

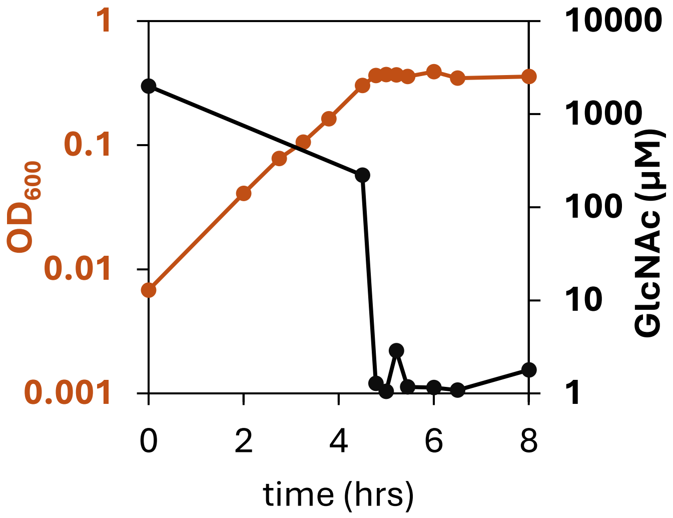

Figure S11: Growth of Vib1A01 in GlcNAc batch culture. A batch culture of *Vib*1A01 growing exponentially in GlcNAc is transferred to a fresh batch culture with 2mM GlcNAc at time 0 and monitored over the next 8 h. OD_{600} of this culture (orange) increased exponentially before halting abruptly at ~0.3, where GlcNAc concentration (black) in the medium dropped to the order of $1\mu M$, and remained there the next several hours where the OD is saturated. The result indicates that the K_M of *Vib*1A01 for GlcNAc is in the range of $1\mu M$.

2. Cell growth and chitinase synthesis.

We now apply the above considerations to the growth of chitin degraders and exploiters. The growth equations for the degraders and exploiters are respectively

$$\frac{d}{dt}A = r_A(n_s) \cdot A - \delta_A A \qquad \text{(N3.2.1)}$$

$$\frac{d}{dt}C = r_C(n_s) \cdot C - \delta_C C \qquad \text{(N3.2.2)}$$

where the 2nd terms on the right hand side of Eqs. (N3.2.1), (N3.2.2) describe cell detachment from chitin particles, with δ_i being the detachment rate of species i. The detachment of *Vib*1A01 is well established experimentally[6]. A similar term is included for the exploiters. Also, we assume the detached cells do not find their way back to the chitin particles. [Reattachment was already small for chitin chips, and is expected to be even smaller for the much smaller colloidal chitin particles.]

Lastly, the equation describing the synthesis of chitinases by the degrader is

$$\frac{d}{dt}E = \varphi_E r_A(n_s)A - \delta_E \cdot E \quad \text{(N3.2.3)}$$

where we assume the chitinase production flux is a constant fraction φ_E of biomass production flux $r_A \cdot A$ (see Guessous et al.[6]), and δ_E describes the rate of chitinase loss (either detachment or turnover, also established in Guessous et al.[6]).

Using Eq. (N3.1.7) for $r_A(n_s)$, Eqs. (N3.2.1)-(N3.2.3) become

$$\frac{d}{dt}A = \kappa_E Y E(t)\frac{A}{A(t) + \eta C(t) + \rho_{leak}} - \delta_A A \quad \text{(N3.2.4)}$$

$$\frac{d}{dt}C = \kappa_E Y E(t)\frac{\eta C}{A(t) + \eta C(t) + \rho_{leak}} - \delta_C C \quad \text{(N3.2.5)}$$

$$\frac{d}{dt}E = \varphi_E \kappa_E Y E(t)\frac{A}{A(t) + \eta C(t) + \rho_{leak}} - \delta_E E \quad \text{(N3.2.6)}$$

By defining an important effective parameter

$$\mu \equiv \varphi_E \kappa_E Y \quad \text{(N3.2.7)}$$

and re-expressing $E(t)$ as $\mathcal{E}(t) \equiv E(t)/\varphi_E$, we obtain a more transparent set of equations:

$$\frac{d}{dt}A = \mu\mathcal{E}(t)\frac{A}{A(t) + \eta C(t) + \rho_{leak}} - \delta_A A \quad \text{(N3.2.8)}$$

$$\frac{d}{dt}C = \mu\mathcal{E}(t)\frac{\eta C}{A(t) + \eta C(t) + \rho_{leak}} - \delta_C C \quad \text{(N3.2.9)}$$

$$\frac{d}{dt}\mathcal{E} = \mu\mathcal{E}(t)\frac{A}{A(t) + \eta C(t) + \rho_{leak}} - \delta_E \mathcal{E} \quad \text{(N3.2.10)}$$

3. Analysis of systems dynamics

We first investigate a possible exponential growth phase admitted by Eqs. (N3.2.8)-(N3.2.10). From the forms of Eqs. (N3.2.8)-(N3.2.10), we see that if the density of the exploiters $C(t)$ increases faster than that of the degraders, $A(t)$, then the factor $A/(A + \eta C + \rho_{leak})$ will diminish over time and an exponential growth phase is not possible. We thus look for the exponential growth phase with $\mathcal{E}(t) = \mathcal{E}_0 e^{\lambda t}$, $A(t) = A_0 e^{\lambda t}$, and $C(t) = C_0 e^{\lambda_C t}$, with the population growth rate for exploiters, λ_C , being slower than that of the degraders, λ. Then Eqs. (N3.2.8)-(N3.2.10) lead to

$$\lambda = \mu - \delta_E \quad \text{(N3.3.1)}$$

$$\frac{\mathcal{E}_0}{A_0} = \frac{\lambda + \delta_A}{\mu} \quad \text{(N3.3.2)}$$

$$\eta < \frac{\delta_C + \mu - \delta_E}{\delta_A + \mu - \delta_E} \quad \text{(N3.3.3)}$$

As $\eta \approx 1$ empirically (see **Supp Note 2**), condition (N3.3.3) is satisfied if the exploiters detach or turn over with a rate similar to that of the degraders. And given that the exploiter growth must be sub-leading in an exponentially growing culture, we shall focus on the dynamics of the degraders and the chitinases in the remainder, taking into account of the exploiters only through their effect on shifting ρ_{leak}. Thus, we focus on the two-variable system

$$\frac{d}{dt}A = \mu\mathcal{E}(t)\frac{A}{A + A_M} - \delta_A \cdot A \quad \text{(N3.3.4)}$$

$$\frac{d}{dt}\mathcal{E} = \mu\mathcal{E}(t)\frac{A}{A + A_M} - \delta_E \cdot \mathcal{E} \quad \text{(N3.3.5)}$$

where

$$A_M \equiv \rho_{leak} + \eta C_0 \quad \text{(N3.3.6)}$$

C_0 being the initial exploiter density. To simplify the two-variable system further, we write Eqs. (N3.3.4) and (N3.3.5) in terms of the dimensionless variables, $a(t) \equiv A(t)/A_M$ and $\varepsilon(t) \equiv \mathcal{E}(t)/A_M$. We obtain the following pair of equations

$$\frac{d}{dt}a = \mu\varepsilon \cdot \frac{a}{a+1} - \delta_A \cdot a \quad \text{(N3.3.7)}$$

$$\frac{d}{dt}\varepsilon = \mu\varepsilon \cdot \frac{a}{a+1} - \delta_E \cdot \varepsilon \quad \text{(N3.3.8)}$$

characterized by 3 parameters: μ, δ_A, δ_E.

We next characterize the conditions that enable Eqs. (N3.3.7) and (N3.3.8) to admit the exponentially growing solution. First, we note that if the initial enzyme concentration is zero (i.e., $\varepsilon(0) \equiv \varepsilon_0 = 0$), then no growth is possible. This reflects the simplifying assumption we took in writing down Eq. (N3.2.3), that the chitinase production flux is proportional to the cell replication rate. Since cell replication depends on GlcNAc generated by chitinases, growth is not possible without chitinases to get the system started. In more realistic models, degraders must be able to synthesize some chitinases before generating substantial GlcNAc for uptake. This synthesis can be fueled by cellular carbon storage such as glycogen, converted from the degradation product of biomass components (e.g., the ribosomes, proteins, or lipids), or derived from the assimilation of residual GlcNAc monomers in the colloidal chitin culture. In this work, we do not model this initial dynamical phase, which requires deeper understanding of the cells' physiology during starvation. Instead, we simply express the end result of this initial phase by an "initial" enzyme amount, $E_0 > 0$, or $\varepsilon_0 > 0$ in the dimensionless variable.

With $\varepsilon_0 > 0$ (and a finite initial degrader concentration $a_0 \equiv a(0) > 0$), it is not always the case that the growth solution is obtained. From Eq. (N3.3.8), no growth is possible if $\mu < \delta_E$, i.e., the enzyme reproduction rate must exceed the enzyme loss rate. Restricting to $\mu > \delta_E$, it is easy to see that if

either δ_A or δ_E is zero, then exponential growth occurs for all $a_0 \equiv a(0) > 0$ and $\varepsilon_0 \equiv \varepsilon(0) > 0$: According to Eq. (N3.3.7), if $\delta_A = 0$, then $a(t)$ always increases for all $a_0 > 0$ and $\varepsilon_0 > 0$. As $a(t) \gg 1$, $\varepsilon(t)$ would grow exponentially according to Eq. (N3.3.8) as long as $\mu > \delta_E$, and the exponential increase of ε in turn drives the exponential increase of $a(t)$. Alternatively, if $\delta_E = 0$, then $\varepsilon(t)$ will grow according to Eq. (N3.3.8) for all $a_0 > 0$ and $\varepsilon_0 > 0$; and when $\varepsilon(t) > \delta_E/\delta_A$, $a(t)$ would grow as well, resulting in the exponential growth of $\varepsilon(t)$ and hence $a(t)$. If both $\delta_A > 0$ and $\delta_E > 0$, then the growth phase $\varepsilon \to \infty$ and $a \to \infty$ is not always obtained for all initial conditions $a_0 > 0$ and $\varepsilon_0 > 0$. A separatrix $a_c(\varepsilon)$ separates the set of initial conditions that lead to exponential growth from those that do not, as shown by the red line in **Fig. S12**. For the monoculture ($C = 0$), crossing the red line is just the Allee transition.

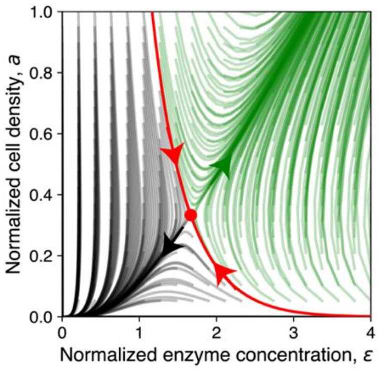

Figure S12: 2-dimensional flow diagram generated by the two-variable dynamics Eqs. (N3.3.7) and (N3.3.8), showing how the cell and enzyme densities (a and ε, respectively) evolve in time. Trajectories are color-coded based on whether they result in growth (green lines) or extinction (gray lines). The red line indicates the separatrix $a_c(\varepsilon)$. The shape of this flow diagram only depends on two effective parameters, δ_A/μ and δ_E/μ, which fix the position of the fixed point (ε^*, a^*) indicated by the red circle. For this plot, we used $\delta_A/\mu = 1$ and $\delta_E/\mu = 0.2$, using parameter values derived from **Table S5**.

The scale of the separatrix, i.e., the condition for which Allee transition occurs, is set by the values of the fixed point, a^* and ε^*, at which $\frac{d}{dt}a = 0$ and $\frac{d}{dt}\varepsilon = 0$. We have $a^* = \delta_E/(\mu - \delta_E)$ and $\varepsilon^* = \delta_A/(\mu - \delta_E)$, which, in terms of the actual cell and chitinase densities, are:

$$E^* = \frac{\delta_A}{\mu - \delta_E}\varphi_E \cdot (\rho_{leak} + \eta C_0) \qquad \text{(N3.3.9)}$$

$$A^* = \frac{\delta_E}{\mu - \delta_E} \cdot (\rho_{leak} + \eta C_0) \qquad \text{(N3.3.10)}$$

Thus, in order for this system to exhibit a transition between the growth and no growth phase, not only must there be the conditions $\mu > \delta_E > 0$, and $\delta_A > 0$, we must also have $\rho_{leak} + \eta C_0 > 0$, i.e., nutrient loss (by diffusive leak or by stealing by exploiters) is *necessary* for a phase transition. Indeed, the scale of the phase transition as set by A^* and E^* is directly proportional to $\rho_{leak} + \eta C_0$. For a system with large ρ_{leak} (such as the case for small colloidal chitin particles studied here), it is possible that the presence of a small amount of exploiters can shift the system from one side of the phase boundary to the other side, resulting in drastic change in the system's fate, from growth to no growth.

4. Numerical solution.

We obtained the separatrix $A_c(E)$ by solving numerically Eqs. (N3.3.4), (N3.3.5) using the parameters listed in **Table S5**. The green line in **Fig. S13A** corresponds to the case of the degrader monoculture with no exploiters, and the grey line in **Fig. S13A** corresponds to a critical initial exploiter density, $C_0 = 0.001$ OD (or 10^6 cells/ml) where the growth of the coculture ceased. To determine the operating

point of the system, we note that the initial degrader density is $A_0 = 0.01$ OD (or 10^7 cells/ml) as used in the experiments.

We next turn to the setting of the initial chitinase concentration, E_0. Molecularly, this is the amount of chitinase carried by the degraders from the previous growth phase, or the amount synthesized by degraders during starvation after they encounter chitin. This amount is fixed within our model by requiring that it poises E_0 for our known condition with $A_0 = 0.01$ OD, near the grey phase transition line. This is shown by the vertical dashed line in **Fig. S13A**, giving $E_0 \approx 2nM$. (This amount of chitinases correspond to ~6.7% of total cellular protein by mass[1]; it is high but plausible given the proteome of *Vib*1A01 [6], or the cell's carbon storage [ref] which can be used for protein synthesis during starvation.)

With these values of (A_0, E_0), we see that the green separatrix falls just below it, such that the system supports exponential growth in the absence of exploiters; but the operating point is on the grey separatrix, such that it does not grow exponentially in the presence of 0.001OD of exploiters. The actual trajectories in the (A, E) phase space is shown as the dashed lines in **Fig. S13B** for $C_0 = 0$ (green), $C_0 = 0.0005$ OD (red), and $C_0 = 0.001$ OD (grey).

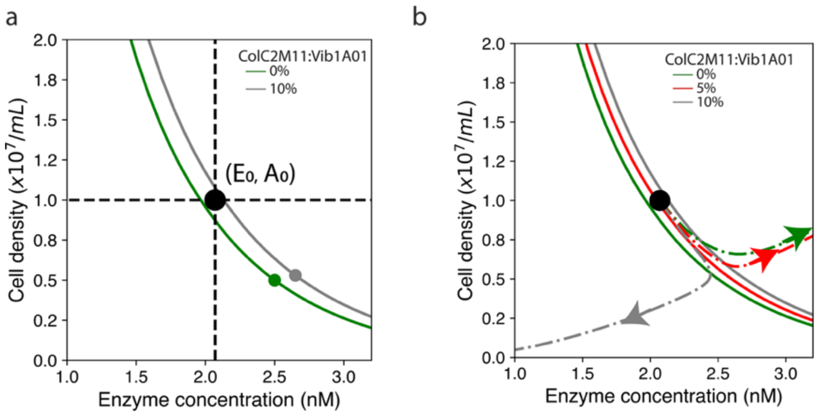

Figure S13: **(A)** Phase diagram in the initial cell and enzyme concentrations. The green line represents the separatrix for the monoculture (C_0: $A_0 = 0$) and the gray line represents the coculture with C_0: $A_0 = 10\%$. The solid green and gray circles represent the fixed points for these respective cases. The operational point (E_0, A_0) is indicated at the crossing of the two dashed lines. **(B)** Trajectories in the A-E space for various initial exploiter densities are indicated as the dashed lines with arrows (colors indicated in the caption).

The time dependences of these 3 cases are shown in **Fig. 6E** of the main text. We find that for the intermediate case with the initial exploiter density being 5% of the initial degrader density (red line in **Fig. 6E**), the increase in lag over the monoculture lag is ~20h, similar to the observation (**Fig. 6F**). Overall, the lag time increases sharply as the critical point is approached (**Fig. S14**), demonstrating

[1] 2nM chitinases at cell density of 0.01 OD is 200 pmol of chitinases per OD*ml of cells. This corresponds to 22 ug for chitinases ~1000 residues in length [refs]. Since each OD*ml of cells contain approximately 330 ug of proteins [guessous23], the initial chitinase amount comprise ~6.7% of the total protein by mass, assuming that the chitinses produced by all cells accumulate on the particles [guessous23]. @Sammy you can manually add ref numbers here.

that this phase transition provides a qualitative metabolic-based mechanism, rationalizing the conundrum that a small increase in exploiter density destroys the growing phase.

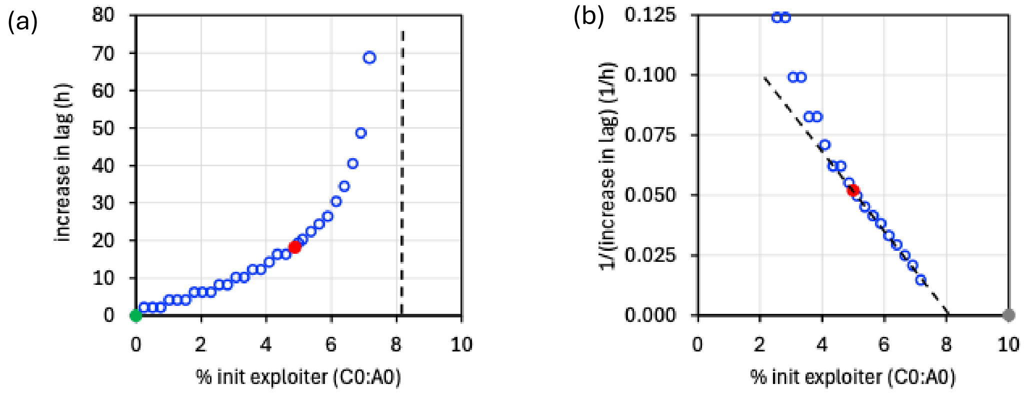

Figure S14: Divergence of lag time at phase transition. (A) Delay of the coculture compared to the monoculture for different initial values of the exploiter, C_0. The green circle represents the monculture and the red circle the coculture with 5% *Col*C2M11 (see **Fig. 6E,F** of the main text). **(B)** Plotting the inverse of the increase in lag reveals the transition for this system occuring at $C_0 : A_0 \approx 8\%$ where the increase in lag diverges. Dashed lines are drawn to guide the eyes.

However, the long lag time of the monoculture exhibited by the model (~48h, **Fig. 6E**) is not consistent with the experimental observations (**Fig. 6F**). The long lag time reflects the difficulty of entering exponential growth. In our model, this may be a result of the simplifying approximation made in **Sec. 1** of this Note, that bacteria cover the surface of the chitin particle uniformly. Such an assumption overlooks the possibility of higher initial bacterial densities on patches of a particle if bacteria are clustered closely together. A more sophisticated treatment would be required to provide a more realistic estimate of the lag time for degraders that start as a surface aggregate.

Further, using our numerical simulations, we are also able to predict the effect of particle size on setting the critical degrader density, A_0, that is required for growth. This requires properly assigning the initial chitinase concentration E_0 for different degrader density, A_0. We assume that E_0 is proportional to the initial degrader density as discussed above, and take the value of the proportionality constant $\alpha \equiv E_0/A_0$ to be 200 nM/OD. With this model of E_0, we first examine the case of the monoculture (i.e., $C_0 = 0$, **Fig. S15**). The strength of the non-linearity of the growth rate resulting from the initial nutrient leakage due to diffusion is exacerbated in the case of small particle sizes. Our model predicts that larger initial degrader densities are required for growth to occur for smaller particle sizes.

Importantly for particle sizes of the order of $R_0 \approx 100\mu m$, the critical density is of the order of $10^{-4} OD$. Such low initial densities are generally difficult to achieve in the laboratory due to the fact that any small amounts of contamination (i.e, dissolved GlcNAc or easily degraded chitin residues) would increase the initial density to this level. In fact, given Vib1A01's yield on GlcNAc, it would only take $0.5\mu M$ to exceed such densities. This analysis explains the difficulty of probing the Allee effect in laboratory conditions, without having to go to very small particle sizes. In our case the combination of both small particle size ($R_0 = 10\mu m$) as well as the additional effect of the degrader allow us to probe this regime. In fact, a similar analysis is performed in the case of the coculture (**Fig. 6J**),

showing that exploiters significantly enhance this effect, especially at densities that correspond to those in the ocean.

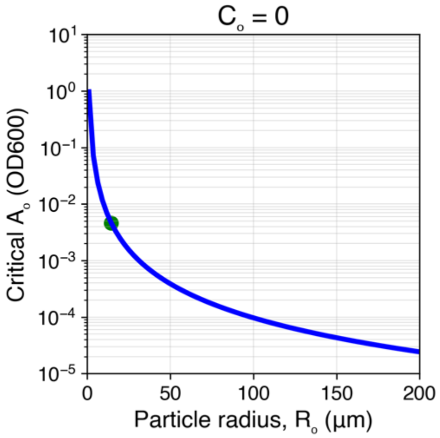

Figure S15: Dependence of critical initial degrader density on particle size. The green dot indicates the experimental condition $R_0 = 10\mu m$.

Parameter description	Symbol	Value	Unit	Reference
GlcNAc diffusion coeff.	D_n	500	$\mu m^2/s$	
colloidal chitin radius	R_0	10	μm	Figure 4
chitin volume fraction	ϕ_{chitin}	0.14	%v/v	Methods
nutrient leakage rate	γ	76	h^{-1}	Eq. (N3.1.6)
*Vib*1A01 cell density	A	0.01-1	OD_{600}; 10^9 cell/ml	footnote a
yield on GlcNAc	Y	0.16	OD_{600}/mM GlcNAc	Refs. [6–8]
max *Vib*1A01 replication rate	$r_{max,A}$	0.6	h^{-1}	Figure S5
max *Col*C2M11 replication rate	$r_{max,C}$	0.3	h^{-1}	Figure S5
*Vib*1A01 Monod constant	K_A	1	μM	Ref [6], Fig. S11
*Col*C2M11 Monod constant	K_C	0.4	μM	footnote b
ratio of the growth rate of *Col*C2M11 and *Vib*1A01 at low GlcNAc concentrations	$\eta \equiv \frac{r_{max,C}/K_C}{r_{max,A}/K_A}$	1.2	--	footnote c, Supp Note 2
population growth rate	λ	0.16	h^{-1}	Figure 6F
chitinase amount per total cellular proteins during exponential growth	φ_E	5%	--	Ref. [6]
chitinase catalytic rate	κ_E		$\frac{\text{mM GlcNAc/h}}{\text{enzyme/mL}}$	footnote d
chitinase regen. rate	$\mu \equiv \varphi_E \kappa_E Y$	0.2	h^{-1}	footnote e Eq. (N3.3.1)
*Vib*1A01 detachment rate	δ_A	0.2	h^{-1}	Ref. [6]
chitinase turnover rate	δ_E	0.04	h^{-1}	Ref. [6]
cell density whose uptake is equivalent to diffusive leakage flux	$\rho_{leak} \equiv \frac{\gamma K_A Y}{r_{max,A}}$	0.02	OD_{600}	Eq. (N3.1.8)
initial *Vib*1A01 density	A_0	0.01	OD_{600}	Figure 1D, 6F
initial *Col*C2M11 density	C_0	0-0.01	OD_{600}	Figure 1D, 6F
initial enzyme concentration for initial degrader density being 0.01 OD	E_0	2	nM	Figure S13
proportionality between the initial enzyme concentration and initial degrader density	$\alpha = E_0/A_0$	200	nM/OD	Figure S13

Table S5: Summary of parameters used in the numerical simulation.

a, In this simulation study, cell density is measured in unit of OD_{600} which is taken to be a density of $10^9\ cells/mL$ for both *Vib*1A01 and *Col*C2M11.

b, The value of the Monod constant for *Col*C2M11 has not been measured and is not needed for the numerical solution. But its value can be inferred from the parameter η, whose value is given in the

next row, together with the knowledge of the parameters $r_{max,A}$, $r_{max,C}$, and K_A listed in rows above. We find $K_C \approx 0.4\ \mu M$.

c, The parameter η, which is the ratio of the growth rate of *Col*C2M11 and *Vib*1A01 at low GlcNAc concentrations and is given by the ratio of the Monod parameters, is obtained from the result of fed-batch growth as described in **Supplementary Note 2**.

d, The value of the catalytic rate of the chitinase, κ_E, was quantified in Guessous et al.[6] for chitin chips. We expect it to be different (larger) for colloidal chitin studied here. The precise value of κ_E is not needed for the model, which depends on κ_E only through the lumped parameter $\mu \equiv \varphi_E \kappa_E Y$, whose value is provided in the next row. [From the values of μ, φ_E, and Y provided, the value of κ_E for colloidal chitin can be inferred and it is 2-3x larger than that found for chitin chips.]

e, For the growing culture, the population growth rate $\lambda \approx 0.16/h$ (**Fig. 6F**) is related to the chitinase regeneration rate μ and the chitinase turnover rate δ_E by Eq. (N3.3.1), $\lambda = \mu - \delta_E$, which gives $\mu \approx 0.2/h$. Note that the population growth rate λ is several times larger here for colloidal chitin compared to that obtained for chitin chips in Guessous et al.[6] This is attributed molecularly to a larger chitinase activity κ_E reflecting the ease of breaking down the chemically treated colloidal chitin.

1. Alvarez, E., Cantoral, J. M., Barredo, J. L., Díez, B. & Martín, J. F. Purification to homogeneity and characterization of acyl coenzyme A:6-aminopenicillanic acid acyltransferase of Penicillium chrysogenum. *Antimicrob Agents Chemother* **31**, 1675–1682 (1987).
2. Hugo, W. & Russel, A. Action of 6-aminopenicillanic acid on gram-negative bacteria. *Nature* (1960).
3. Rolinson, G. & Stevens, S. 6-Aminopenicillanic acid IV. Antibacterial activity. *Nature* (1961).
4. Granato, E. T., Meiller-Legrand, T. A. & Foster, K. R. The Evolution and Ecology of Bacterial Warfare. *Current Biology* **29**, R521–R537 (2019).
5. Pontrelli, S. *et al.* Metabolic cross-feeding structures the assembly of polysaccharide degrading communities. *Science Advances* **8**, 1–12 (2022).
6. Guessous, G. *et al.* Inherited chitinases enable sustained growth and rapid dispersal of bacteria from chitin particles. *Nat Microbiol* **8**, 1695–1705 (2023).
7. Amarnath, K. *et al.* Stress-induced metabolic exchanges between complementary bacterial types underly a dynamic mechanism of inter-species stress resistance. *Nature Communications* **14**, 3165 (2023).
8. Iffland-Stettner, A. *et al.* A Genome-Scale Metabolic Model of Marine Heterotroph Vibrio splendidus Strain 1A01. *mSystems* **8**, e00377-22 (2023).
